# IFN-γ Stimulates Autophagy-Mediated Clearance of *Burkholderia cenocepacia* in Human Cystic Fibrosis Macrophages

**DOI:** 10.1371/journal.pone.0096681

**Published:** 2014-05-05

**Authors:** Kaivon Assani, Mia F. Tazi, Amal O. Amer, Benjamin T. Kopp

**Affiliations:** 1 Center for Microbial Pathogenesis, The Research Institute at Nationwide Children’s Hospital, Columbus, Ohio, United States of America; 2 Department of Microbial Infection and Immunity and the Department of Internal Medicine, The Ohio State University, Columbus, Ohio, United States of America; 3 Section of Pediatric Pulmonology, Nationwide Children’s Hospital, Columbus, Ohio, United States of America; University of Duisburg-Essen, Germany

## Abstract

*Burkholderia cenocepacia* is a virulent pathogen that causes significant morbidity and mortality in patients with cystic fibrosis (CF), survives intracellularly in macrophages, and uniquely causes systemic infections in CF. Autophagy is a physiologic process that involves engulfing non-functional organelles and proteins and delivering them for lysosomal degradation, but also plays a role in eliminating intracellular pathogens, including *B. cenocepacia*. Autophagy is defective in CF but can be stimulated in murine CF models leading to increased clearance of *B. cenocepacia,* but little is known about autophagy stimulation in human CF macrophages. IFN-γ activates macrophages and increases antigen presentation while also inducing autophagy in macrophages. We therefore, hypothesized that treatment with IFN-γ would increase autophagy and macrophage activation in patients with CF. Peripheral blood monocyte derived macrophages (MDMs) were obtained from CF and non-CF donors and subsequently infected with *B. cenocepacia*. Basal serum levels of IFN-γ were similar between CF and non-CF patients, however after *B. cenocepacia* infection there is deficient IFN-γ production in CF MDMs. IFN-γ treated CF MDMs demonstrate increased co-localization with the autophagy molecule p62, increased autophagosome formation, and increased trafficking to lysosomes compared to untreated CF MDMs. Electron microscopy confirmed IFN-γ promotes double membrane vacuole formation around bacteria in CF MDMs, while only single membrane vacuoles form in untreated CF cells. Bacterial burden is significantly reduced in autophagy stimulated CF MDMs, comparable to non-CF levels. IL-1β production is decreased in CF MDMs after IFN-γ treatment. Together, these results demonstrate that IFN-γ promotes autophagy-mediated clearance of *B. cenocepacia* in human CF macrophages.

## Introduction

Cystic fibrosis (CF) is an inherited, life-limiting disease that causes multi-organ dysfunction characterized by progressive respiratory infections with inspissated mucous [1], [2]. Patients with CF can be infected by a variety of pathogens, including the rapidly transmissible *Burkholderia cenocepacia* [3]–[6]. *B. cenocepacia* is a unique CF pathogen that causes either a distinct clinical phenotype of systemic fatal septicemia or hastened chronic respiratory deterioration with diminished long term survival [7], [8]. Therapeutic options are severely limited due to multi-drug resistance and near universal exclusion from lung transplant eligibility due to poor post-transplant survival in chronically infected patients [9]–[13].

Macrophages are a first-line defense against pathogens including *B. cenocepacia.* The vital role of macrophages in CF pathogen interactions, in addition to airway epithelial cells, has been highlighted by several groups [14]–[19]. Bacteria survive in CF macrophages despite successful phagocytosis due to links between CF transmembrane conductance regulator (CFTR) dysfunction and impaired phagolysosomal killing [17], [20], [21]. *B. cenocepacia* is also specifically able to evade degradation in CF macrophages leading to severe and persistent inflammation [19], [22], [23]. Additionally, in model systems *B. cenocepacia* replicates inside of macrophages prior to dissemination [24].

In conjunction with macrophage defects, CF leads to deficient autophagy through inflammatory mediated cross-linking of the essential beclin-1 autophagy initiator interactome [25]. Autophagy is a physiologic process that normally augments innate responses to intraphagosomal pathogens and may relate to macrophage clearance defects. Deficient autophagy prevents destruction of engulfed *B. cenocepacia* in murine CF macrophages [22], [26]. Autophagy stimulation by rapamycin reduces murine CF bacterial burden and inflammation [22]. Autophagy stimulation with rapamycin also enhances clearance of other major CF pathogens such as *Pseudomonas aeruginosa* [27] and *Staphylococcus aureus* [28], but autophagy has not been studied in human CF macrophages. Restoration of functional autophagy with an anti-inflammatory peptide, IDR-1018, decreases inflammation in CF cells [29], and may globally alleviate CF symptoms by releasing sequestered essential autophagy molecules, such as the autophagy adaptor molecule P62/SQSTM1 (p62)[30]. Relief of sequestered autophagy molecules enabled protein aggregate clearance and improved CFTR trafficking, lending benefits of autophagy-stimulated pathogen clearance [25].

Although rapamycin was effective in murine CF studies, systemic side effects of rapamycin in CF [31] and increased development of interstitial lung disease [32] prevent clinical trials. Additionally, autophagy stimulation by immununologics, such as rapamycin, raises questions how this would affect other microorganisms in the lungs of CF patients. Alternatively, IFN-γ has been shown to increase macrophage clearance of apoptotic cells in chronic granulomatous disease (CGD) [33], and is used prophylactically in CGD patients to prevent acquisition of severe infections such as *Burkholderia* species [34], [35]. CGD, the only other known clinical disease commonly affected by *Burkholderia* species, also has known phagocytic killing defects [36], [37] and was recently shown to have deficient autophagic responses to *B. cenocepacia* [*38*]. Furthermore, IFN-γ treatment enhances the autophagy response in macrophages derived from non-CGD patients resulting in clearance of pathogens that evade typical phagocytic degradation [39]. IFN-γ therapy is well tolerated in CGD patients who have altered immune functions and polymicrobial infections similar to CF [34], [40]. Although a trial of inhaled IFN-γ in CF patients with mild to moderate disease did not prove effective in altering lung function [41], IFN-γ has never been studied systemically in CF, or specifically in *B. cenocepacia* infected patients, who may require more than local lung treatment.

We hypothesize that due to known defects in CF macrophage autophagy, IFN-γ will reduce *B. cenocepacia* burden in human CF macrophages through more effective killing and enhanced autophagy that degrade *B. cenocepacia* in nascent vacuoles.

## Materials and Methods

### Bacterial Strains and Culture

*Burkholderia cenocepacia* strain k56-2 is a clinical isolate of the ET12 lineage originally isolated from a CF patient’s sputum. The strain was tagged dsRED and grown in Luria-Bertani (LB) broth at 37°C overnight with high amplitude shaking. The *B. cenocepacia* MHK1 strain is derived from k56-2 and has a mutation in an antibiotic efflux pump that confers gentamicin sensitivity, but does not alter the trafficking of the mutant in macrophages [42]. For colony forming unit (CFU) analysis, 50 µg/ml gentamicin (Invitrogen, 3564) was added for 0.5 hours as described previously [43]. To enumerate intracellular bacteria, infected macrophages were lysed with ice-cold PBS (Invitrogen, 14190) at designated times. Extracellular bacteria were enumerated directly from cell supernatants during macrophage viability assays. Recovered bacteria were quantified by plating serial dilutions on LB agar plates and counting colonies using the Acolyte Colony Counter, 5710/SYN.

### Human Monocyte-Derived Macrophages (MDMs) –Ethics Statement

Human subjects underwent written informed consent for blood donation at the Nationwide Children’s Hospital as approved by the Institutional Review Board of Nationwide Children’s Hospital. Written consent from legal guardians of minors was obtained as well as written assent from minors aged 9 to 17 years. Blood was drawn in heparinized tubes. Exclusion criteria included history of *B. cepacia* complex culture positivity, chronic immunosuppression, CFTR modulator use, and history of transplantation. Chronic azithromycin was the only immunomodulatory medicine taken by some subjects during the study period. Peripheral blood mononuclear cells (PBMCs) were isolated from 27 CF and 27 non-CF healthy controls. Monocytes were separated using a Ficol gradient via Lymphocyte Separation Medium (Corning, 25-072-CV). Isolated monocytes were re-suspended in RPMI (Gibco, 22400-089) and 10% human AB serum (Lonza, 14-490E) to a concentration of 2×10^6^ cells/mL and incubated for 5 days at 37°C to derive macrophages. Macrophages were then infected with *B. cenocepacia* strain k56-2 at a multiplicity of infection (MOI) ranging from 2–10 over the stated times.

### Reagents

IFN-γ (eBioscience, 14-8319-80) was used at a concentration of 200 ηg/ml based upon effectiveness in trial experiments. Rapamycin (LC laboratories, AY-22989) was added to the cells at a concentration of 25 µg/ml. Treatments were added 1 hour post infection and the cells were allowed to incubate for 2, 4, and 24hours before obtaining results. Two mM 3-methyladenine (3MA, M9281) and 10 nM Bafilomycin (Sigma-Aldrich, B1793) were added one hour prior to infection in inhibitor experiments.

### Immunoblotting

Macrophage supernatants were removed post treatment and the cells were washed twice with phosphate buffered saline (PBS). The cells were lysed in lysis buffer and primed with a protease inhibitor (Roche Applied Science, 10-519-978-001). Then, 30 ug of protein was separated by SDS-PAGE and transferred onto polyvinylidene difluoride (PVDF) membranes. Membranes were immunoblotted for LC3 (Sigma-Aldrich, L8918), p62 (Sigma-Aldrich, P0067), beclin-1 (Abcam, ab51031), actin (Abcam, ab8226-100), IFNGR1 (Abcam, ab61179), IFNGRbeta (Abcam, ab84524), and calreticulin (Stressgen, #SPA-600). Protein bands were detected with HRP-conjugated secondary antibodies visualized using enhanced chemiluminescent (ECL) reagents (Life Sciences, RPN2106).

### Enzyme-Linked Immunosorbent Assay (ELISA)

MDM culture supernatants were clarified and stored at −20°C until assayed for cytokine content. MDMs were infected for 4 and 24 hours with k56-2. The quantification of IL-1β, IFN-γ, and IL-10 in supernatants was determined by sandwich ELISA following the manufacturer’s protocol (R&D system Inc, DY285) as previously described [43].

### Cytotoxicity

MDMs were infected with k56-2 for 4 and 24hours and the culture supernatants were collected and centrifuged. Histone-associated DNA fragments were detected using a cytotoxicity detection photometric assay kit according to the manufacturer’s protocol (Roche Applied Science, 11 644 793 001). All experiments were performed in at least triplicate. Additionally, macrophage viability was assessed via naphthol staining. MDMs were plated in 24-well plates, infected for 1 h, then treated for 24 h. Cells were washed and treated with 1% Cetavlon in 0.1 M citric acid with 0.05% Napthol blue black (Sigma-Aldrich), pH 2.2, for 15 min at room temperature. Stained nuclei were enumerated on a haemacytometer using phase-contrast microscopy.

### Confocal Microscopy

Confocal microscopy sample were analyzed with an Olympus FV10i Spectral Confocal microscope. Two million MDMs were cultured on 12 mm glass cover slips in 24-well tissue culture plates and infected synchronously with k56-2 at an MOI of 2 or 10. Nuclei were stained with the nucleic acid dye 4′,6′-diamino-2-phenylindole (DAPI) blue for imaging. LC3 stained green with a cleaved LC3 antibody detection (Abgent, AP1805a). Lysosomes were stained green with Lysotracker Green (Invitrogen, L7526). p62 was detected with a green fluorescent ligand (BD Bioscience, 610832). At least one hundred macrophages were scored for each condition with scoring verified by independent study members. All experiments were performed in at least triplicate.

### Transmission Electron Microscopy

TEM Images were obtained using a FEI Technai G2 Spirit transmission electron microscope (FEI, USA), Macrofire (Optronics) digital camera and AMT image capture Software with assistance from the Campus Microscopy and Imaging Facility (CMIF) at The Ohio State University. MDMs were isolated and infected with k56-2 at an MOI of 10 for 1 hour prior to 24 hour experimental treatments. Cells were cultured on Permanox (Lab-Tek) chamber slides and fixed with 2.5% gluteraldehyde in 0.1 M phosphate buffer with 0.1 M sucrose. Slides were post fixed with 1% osmium tetroxide in phosphate buffer then en bloc stained with 2% uranyl acetate in 10% ethanol, dehydrated in a graded series of ethanols and embedded in Eponate 12 epoxy resin (Ted Pella Inc., USA). Ultrathin sections were cut on a Leica EM UC6 ultra microtome (Leica microsystems, Germany), collected on copper grids, and then stained with lead citrate and uranyl acetate.

### Statistical Analysis

Statistical analysis was performed using GraphPad Prism software (version 6.0). Statistical significance was determined with a two-tailed *p*<0.05. Mann-Whitney was used for non-parametric measurements, and ANOVA was used where appropriate.

## Results

### Patient Demographics

Subject demographics are described in Table 1. CF and non-CF subjects were similar in terms of ethnicity (100% Caucasian) and mean age (30.4±11.5 years vs. 33.5±9.2 years, p = 0.53). Subjects with CF had a mean forced expiratory volume in one second (FEV1) % predicted of 57.1%, indicative of moderately severe lung function at the time of sampling. 81% of CF patients had chronic respiratory cultures positive for *P. aeruginosa,* and 48% had methicillin resistant *S. aureus* (MRSA). Importantly, 48% of subjects were on chronic azithromycin prophylaxis, and this treatment was not noted to influence the experiments below. Azithromycin has been show to block autophagy in CF macrophages in vitro [44], but CF Patient Registry data does not support an increased risk of mycobacterial infections for those on chronic azithromycin as proposed in the study by Renna and colleagues[45]. Cell cultures in our experiments were not further treated with azithromycin.

**Table 1:** Patient Demographics.

	non CF patients (n = 27)	CF patients (n = 27)
Mean age (years ± st. dev.)	33.5±9.2	30.4±11.5
Males	30%	59%
Caucasian	100%	100%
Mean FEV1 (% predicted)	N/A	57.1±21.4
Pseudomonas colonization	N/A	81%
MRSA colonization	N/A	48%

### IFN-γ Production is Reduced in CF PBMCs

**Figure 1:**
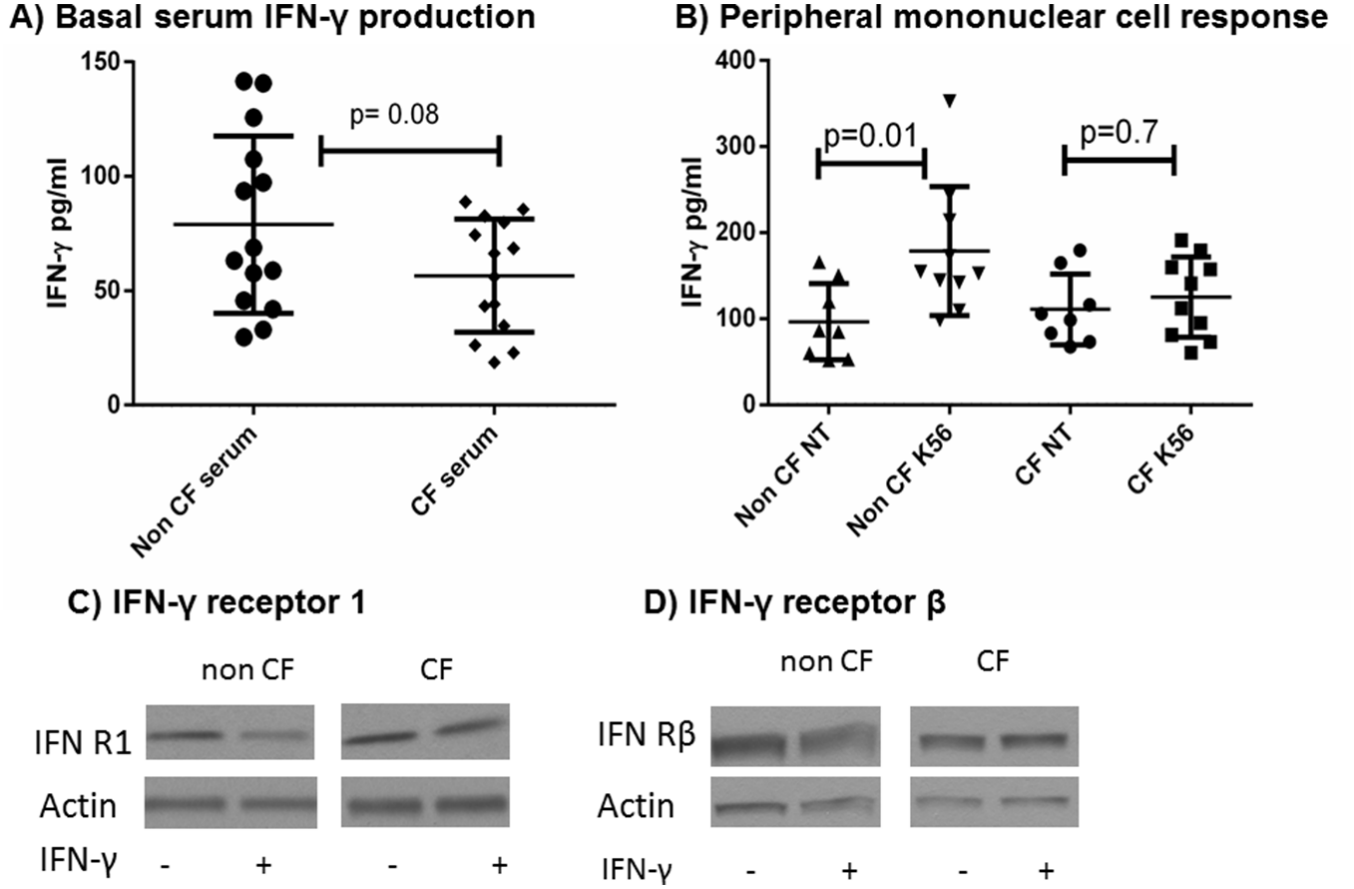
IFN-γ is deficiently produced in CF PBMCs in response to *B. cenocepacia.* 1A) IFN-γ levels from the serum of 14 non-CF and 14 CF patients at the time of blood donation, p = 0.056, n = 14 subjects for each condition, Mann-Whitney testing. 1B) IFN-γ production from PBMC 24 hour cell supernatants from CF and non-CF patients. NT represents uninfected cells, and k56 were infected with *B. cenocepacia* k56-2. n = 8 subjects for NT and 10 subjects k56, Mann-Whitney testing. 1C) Immunoblot for IFN-receptor 1 from cell lysates of MDMs infected with k56-2+/− treatment with IFN-γ. 1D) Immunoblot for IFN-receptor β from cell lysates of MDMs infected with k56-2+/− treatment with IFN-γ.

IFN-γ effectively stimulates autophagic responses in macrophages [46], [47]. However, CF cells insufficiently produce IFN-γ in response to another pathogen, *P. aeruginosa* [48]. To determine if IFN-γ is differentially produced in CF in response to *B. cenocepacia*, IFN-γ was measured in the serum of CF and non-CF subjects prior to macrophage isolation and in PBMC culture supernatants with and without 24 hour *B. cenocepacia* infection. Mean serum levels of IFN-γ were non-significantly lower in CF versus non-CF subjects (56.6±6.6 vs. 78.9±10.4, p = 0.08, Figure 1A). Infected non-CF PBMC supernatants displayed significantly higher IFN-γ levels compared to uninfected, whereas there was no change in IFN-γ production between uninfected and infected CF PBMCs (Figure 1B). Uninfected CF and non-CF PBMC IFN-γ levels were both slightly higher than serum levels. There was no difference in IFN-γ production between CF patients on azithromycin therapy and those not (figure S1A). Additionally, there was no difference in IFN-γ receptor expression during infection between IFN-γ treated and non-treated MDMs as measured by immunoblotting (Figures 1C, 1D). Our results are consistent with studies from *P. aeruginosa* [48], suggesting a defective host CF IFN-γ response to multiple pathogens in CF.

### IFN-γ Increases *B. cenocepacia* Co-localization with p62

**Figure 2:**
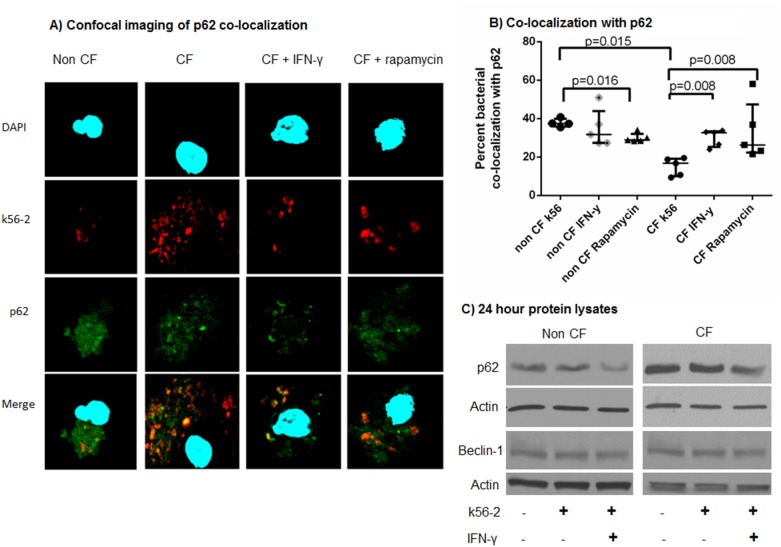
IFN-γ increases *B. cenocepacia* co-localization with p62 and decreases p62 accumulation in CF. 2A) Confocal microscopy for non-CF and CF macrophages infected with m-RFP expressing k56-2. IFN-y or rapamycin treatment was administered after 1 hour of infection for a 24 hour treatment period. p62 is stained green, and macrophage nuclei are stained blue with DAPI. Co-localization of bacteria with p62 is noted in yellow in the bottom panel. 2B) The percentage of bacterial co-localization with p62 was scored for over 100 macrophages per condition, n = 5 subjects per condition, Mann-Whitney testing. 2C) Immunoblot for non-CF and CF macrophages demonstrating p62 accumulation in CF with reduction during IFN-y therapy, representative of 5 subjects. Immunoblot of beclin-1 levels for non-CF and CF macrophages from cell lysates of control (NT) and MDMs infected with k56-2+/− treatment with IFN-γ, n = 4.

p62 is an essential intracellular protein that targets cargo for autophagy as well as regulates signaling pathways involved in cell survival and/or death [49]. This docking molecule is required for targeting *B. cenocepacia* to autophagosomes in a murine model[26], however its mechanism of action in human cells infected with *B. cenocepacia* is unknown. Using confocal microscopy, we investigated how p62 targets *B. cenocepacia* in human CF macrophages by examining MDMs for co-localization of bacteria with p62 after a 24 hour infection. MDMs were stimulated with rapamycin or IFN-γ for the duration of infection. Untreated CF macrophages demonstrated significantly less median co-localization with p62 compared to non-CF macrophages (16.8% (10.1–19.1) vs. 37.0% (34.9–40.0), p = 0.016, Figures 2A, B). IFN-γ or rapamycin treatment significantly increased median bacterial co-localization with p62 in CF human MDMs (CF IFN-γ 32.7% (25.2–33.4), p = 0.006, CF rapamycin 26.3% (22.4–47.4), p = 0.008, Figures 2A,B) nearing non-CF levels. p62 levels detected by western blotting are increased in uninfected and infected CF MDMs compared to non-CF MDMs (Figure 2C). Twenty-four hour IFN-γ treatment of non-CF and CF MDMs resulted in a decrement in p62 accumulation (Figure 2C). To ensure that initiation of the beclin-1 interactome was not influencing p62 expression, beclin-1 was measured via immunoblotting and confocal microscopy after 24 hour infection. There was no difference in beclin-1 levels between CF and non CF MDMs (Figure 2C, confocal not shown). Altogether, this data suggested that p62 is either sequestered or inefficiently utilized by human CF MDMs, and IFN-γ can alleviate this undesirable phenotype in CF cells.

### Autophagic Flux is Enhanced by IFN-γ in CF MDMs

**Figure 3:**
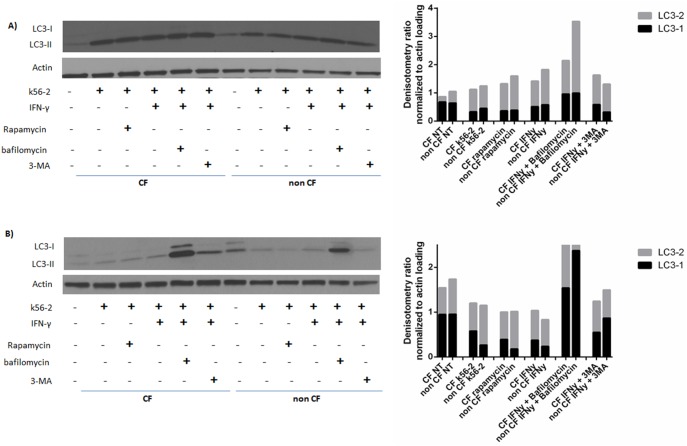
Autophagic flux is decreased in CF. 3A) Representative immunoblot of 4 hour treatment protein lysates for LC3-1 and LC3-2 from non-CF and CF macrophages, with corresponding summed band densitometries normalized to loading control. All lanes are marked with a “+” if the corresponding treatments were added: *B. cenocepacia* infection (k56-2), autophagy stimulators (IFN-y, Rapamycin) and autophagy inhibitors (Bafilomycin, 3-MA). Inhibitors were added one hour prior to infection. Autophagy stimulators were added one hour after infection for a total of 4 hours of treatment. Data is representative of 3 independent experiments. 3B) Representative immunoblot of 24 hour treatment protein lysates for LC3-1 and LC3-2 from non-CF and CF macrophages with corresponding summed band densitometries normalized to loading control. All lanes are marked with a “+” if the corresponding treatments were added: *B. cenocepacia* infection (k56-2), autophagy stimulators (IFN-y, Rapamycin) and autophagy inhibitors (Bafilomycin, 3-MA). Inhibitors were added one hour prior to infection. Autophagy stimulators were added one hour after infection for a total of 24 hours of treatment. Data is representative of 3 independent experiments.

Autophagic flux is deficient in CF cells [25], [30]. Microtubule-associated proteins 1A/1B light chain 3A (LC3) can be monitored to assess autophagosome formation and flux. During the autophagic process, cytosolic LC3 (LC3-I) is conjugated to phosphatidylethanolamine to form LC3-II, which is then recruited to the autophagosomal membrane. We analyzed LC3 concentrations by immunoblotting to monitor the conversion of LC3-I to LC3-II, as well as depletion/accumulation of the proteins to assist in determination of turnover of proteins. During basal, uninfected conditions (NT), CF macrophages demonstrated persistence of increased LC3-1 over 24 hours compared to non CF as evidence by high proportion of LC31 relative to total LC3 (Figures 3A, 3B). Infection with k56-2 leads to persistently elevated LC3-1 levels in CF by 24 hours compared to non CF, but to a lesser extent than basal conditions (Figure 3B). LC31 levels are seen to decrease in CF similar to non CF levels by 24 hours with the use of either IFN-γ or rapamycin (Figure 3B). Specific autophagy inhibitors were used to monitor treatment effects. The addition of bafilomycin to inhibit autophagolysosomal fusion via inhibiting vacuolar H+ ATPase (V-ATPase) caused an increase in LC3-1 in CF at 4 and 24 hours (Figures 3A,3B), reversing the effects of IFN-γ. The addition of 3-methyladenine (3-MA) to block autophagosome formation via inhibiting type III Phosphatidylinositol 3-kinases had a similar, but lesser effect in CF on LC3 conversion compared to bafilomycin. Combining data from 4 and 24 hour time points suggests a deficit in autophagic flux in CF macrophages, with enhancement through autophagy stimulators such as IFN-γ that are increasing autophagosome formation.

### Autophagy Stimulation Increases Bacterial Clearance in CF

**Figure 4:**
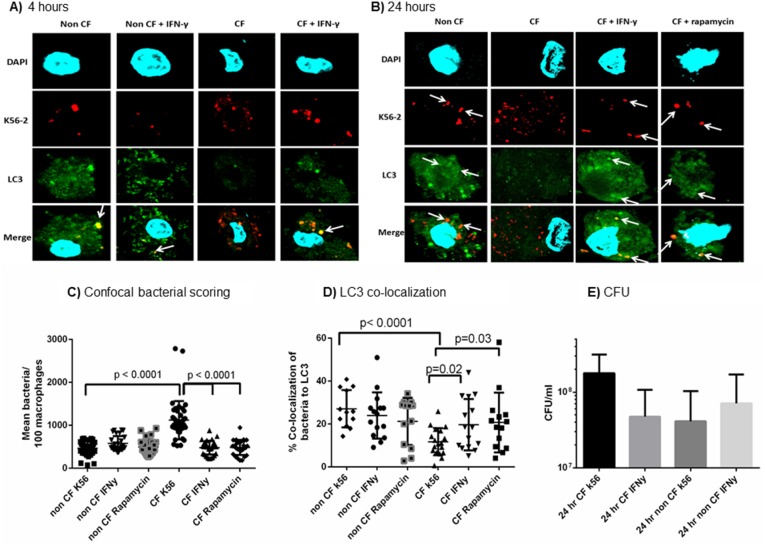
IFN-γ increases autophagosome formation. 4A) Confocal microscopy for non-CF and CF macrophages infected with m-RFP expressing k56-2. IFN-y treatment was administered after 1 hour of infection for a 4 hour treatment period. LC3 is stained green, and macrophage nuclei are stained blue with DAPI. Co-localization of bacteria with LC3 is noted in yellow in the bottom panel as noted by white arrows. 4B) 24 hour IFN-γ and rapamycin treatments similar to 4A. 4C) Summary of scored bacteria per 100 macrophages from individual subjects for the confocal microscopy experiments from 4B, n = 17 subjects, with 2 replicates per subject, unpaired t-test. 4D) The percentage of bacterial co-localization with LC3 was scored for over 100 macrophages per condition from 4B, n = 17 subjects, with 2 replicates per subject, unpaired t-test. 4E) CFU counts for non-CF and CF macrophages infected with MHK1 for 24 hours (n = 6).

**Figure 5:**
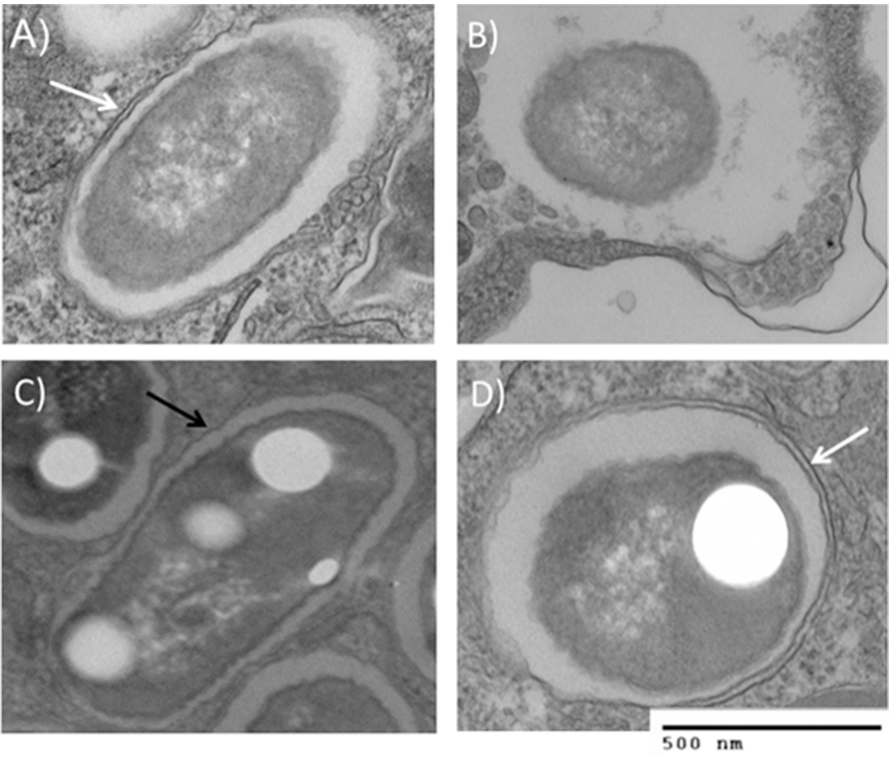
IFN-γ stimulates double-membrane autophagosome formation. 5A) Electron microscopy of non-CF macrophage infected with k56-2 only for 24 hours. White arrow indicates double membrane formation indicative of autophagosomes. 5B) EM of non-CF macrophage treated with IFN-γ for 24 hours. 5C) EM of CF macrophage infected with k56-2 only. Black arrow indicates single membrane vacuole. 5D) EM of CF macrophage treated with IFN-γ for 24 hours. White arrow indicates double membrane formation. Pictures are marked with 500 nm marker.

*B. cenocepacia* infection causes increased bacterial burden in human and murine CF cells, indicating an innate defect in clearing the pathogen in CF [19], [22]. Persistent bacterial loads can lead to systemic involvement and/or heightened inflammation. Because autophagy regulates pathogen clearance, we stimulated autophagy in MDMs with IFN-y or rapamycin for 4 and 24 hours and assessed bacterial clearance by confocal microscopy and CFU. Twenty-four hours is an expected clinical dosing regimen of IFN-γ, and 4 hours was chosen to ensure sufficient autophagosome formation, as murine studies have shown that even in wild type macrophages *B. cenocepacia* requires at least 2 hours to traffic to autophagosomes. There is no difference in bacterial counts at 2 hours (figure S2). At 4 hours of treatment, there were significantly higher bacterial counts in both autophagy-stimulated and unstimulated CF macrophages compared to non-CF macrophages (Figures 4A). However, CF macrophages treated with IFN-γ for 4 hours had a higher proportion of bacterial co-localized with autophagosomes as marked by LC3 compared to untreated CF macrophages (Figure 4A). A 24 hour treatment of IFN-γ or rapamycin markedly reduced bacterial counts in the CF macrophages compared to untreated CF macrophages (Figures 4B, 4C, 4E). These reductions mirror bacterial levels in non-CF macrophages. There was no difference in bacterial counts between CF patients on azithromycin therapy and those not (Supplemental Figure 1B). 24 hour extracellular bacterial counts were not reduced (Figure S2). There is a sustained increase in the co-localization of bacteria with LC3 after 24 hours of autophagy stimulation in CF macrophages compared to untreated CF macrophages (p = 0.02, Figures 4B, 4D). Electron microscopy confirmed these confocal findings. Untreated non-CF macrophages contained double membrane vacuoles surrounding *B. cenocepacia,* indicative of autophagosome formation (Figure 5A). Untreated CF macrophages displayed only single membrane bound vacuoles (5C), but when stimulated with IFN-γ, CF macrophages displayed double membrane vacuoles similar to the non-CF (5D). This observation suggests autophagosome formation is stimulated upon IFN-γ treatment in the CF MDMs. Additionally, IFN-γ had no direct effects on bacterial growth when added to bacteria in media devoid of MDMs, with no difference in bacterial growth over 24 hours between media with k56-2 alone, and media with k56-2 plus IFN-γ (Figure S3). In summation, these results indicate that IFN-γ effectively stimulates early autophagic targeting of *B. cenocepacia* to autophagosomes, thus allowing enhanced clearance after 24 hours.

### Lysosome Targeting is Increased in Autophagy Stimulated CF MDMs

**Figure 6:**
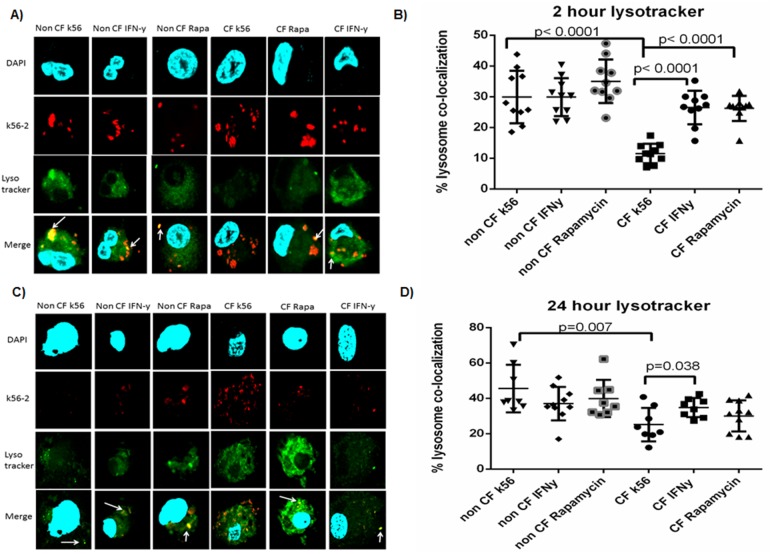
Lysosomal co-localization is increased with IFN-γ in CF. 6A) Confocal microscopy for non-CF and CF macrophages infected with k56-2. Macrophages were labeled with green lysotracker and bacterial co-localization to lysosomes was measured after a 2 hour infection. Macrophage nuclei are stained blue with DAPI. Co-localization of bacteria with lysotracker is noted in yellow by white arrows. 6B) Quantitative scoring of % bacterial co-localization with lysosomes from 6A, n = 8 subjects, Mann-Whitney testing. 6C) 24 hour k56-2 infection with lysotracker staining. 6D) Bacterial co-localization with lysotracker quantitative scoring for 5C, n = 8 subjects, Mann-Whitney testing.

Rapamycin increases lysosomal degradation of *B. cenocepacia* in CF murine macrophages by promoting phagosome-lysosome fusion via autophagy [22]. Human CF macrophages also demonstrate decreased co-localization of *B. cenocepacia* with lysosomes[19]. We examined the extent to which IFN-γ enhances autophagosome fusion with lysosomes by infecting human MDMs with *B. cenocepacia* for 1 hour and then treating with IFN-γ for 2 and 24 hours. We then scored co-localization of a fluorescent lysotracker with bacteria using confocal microscopy. After 2 hours of infection, untreated CF macrophages exhibited very little co-localization of bacteria with lysosomes (11.5±3.2%, Figures 6A, B) compared to non-CF macrophages (29.9±8.5%, Figures 6A, B). However, CF macrophages treated with IFN-γ demonstrate increased lysosomal co-localization of bacteria (26.5±.5%, p<0.0001, Figures 6A, B). After 24 hours of IFN-γ therapy CF macrophages demonstrated a sustained lysosomal co-localization of remaining bacteria, similar to what was observed in non-CF macrophages (34.8±5.4% vs. 37.0±9.5%, p = 0.5, Figures 6C, D). Untreated CF macrophages have significantly less lysosomal co-localization compared to treated CF macrophages at 24 hours. (25.1±9.4%, p = 0.038, Figures 6C, D). Together, the results demonstrate delayed lysosomal targeting of *B. cenocepacia* in CF MDMs. Combined with prior results, IFN-γ treatment increases autophagosome formation in CF macrophages, enhancing lysosomal targeting of *B. cenocepacia*.

### IL-1β Production is Decreased with Autophagy Stimulation

**Figure 7:**
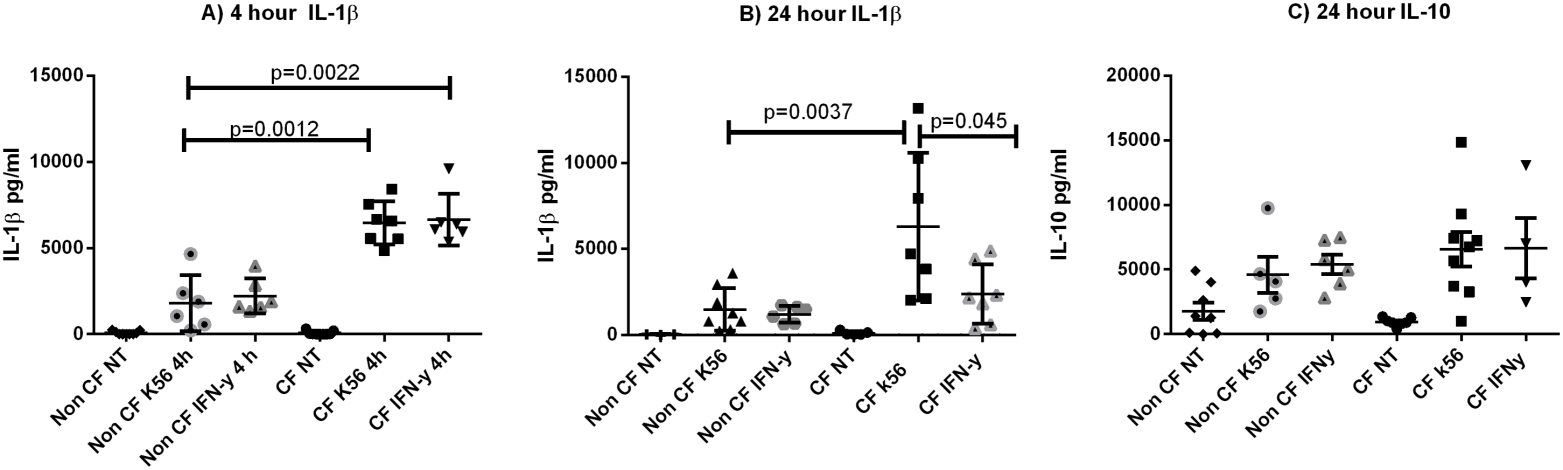
IL-1β is decreased with IFN-γ treatment in CF. IL-1β levels in macrophage supernatants after infection with k56-2 and either a 4 hour treatment with IFN-γ or a control diluent (7A) or a 24 hour IFN-γ treatment (7B), n = 7 subjects, Mann-Whitney analysis. 7C) IL-10 levels in macrophage supernatants after infection with k56-2 and a 24 hour treatment with IFN-γ or a control diluent, n = 7 subjects, Mann-Whitney analysis. Significant differences are noted.

CF macrophages excessively produce the pro-inflammatory cytokine IL-1β in response to *B. cenocepacia* infection [19], [50]. Human bronchial epithelial cells do not produce IL-1β in response to *B. cenocepacia* [51]. During *B. cenocepacia* infection pyrin inflammasome activation results in IL-1β release from mononuclear cells [52]. Therefore, we measured IL-1β production in human CF macrophages after autophagy stimulation as a primary source of excess inflammatory cytokine production during infection. MDMs were infected with *B. cenocepacia* and treated with IFN-γ or rapamycin for 4 and 24 hours and cell supernatants were examined by ELISA for IL-1β production. After 4 hours of autophagy stimulation, IL-1β levels were similar between treated and untreated CF macrophages, but CF macrophages had significantly more IL-1β than non-CF macrophages (p = 0.0012, Figure 7A). However, in CF macrophages treated with IFN-γ, IL-1β levels diminished by 24 hours of treatment (p = 0.045), while untreated CF macrophages infected with *B. cenocepacia* perpetuate elevated IL-1β production (Figure 7B). There was no difference in IL-1β production between CF patients on azithromycin therapy and those not (figure S1C). IL-10 was also measured as it can directly impair IFN-γ signaling pathways and its production can correlate with inflammatory cytokines such as IL-1β. There was no difference in IL-10 production in 24 hour supernatants between uninfected, infected, and IFN-γ treated CF and non-CF macrophages (Figure 7C). These results demonstrate autophagy induction plays a role in reducing IL-1β production in macrophage responses to *B. cenocepacia.*

### Cell Death is Decreased by IFN-γ Treatment during *B. cenocepacia* Infection

**Figure 8:**
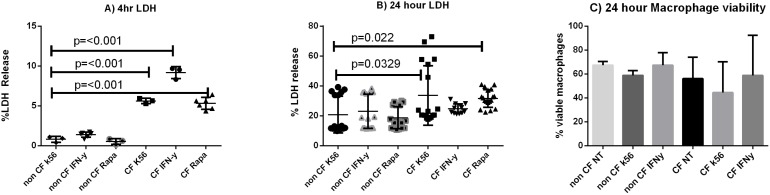
Cell death is decreased with autophagy stimulation in CF. Percent LDH release from MDM supernatants treated with autophagy stimulators IFN-γ or Rapamycin for 4 hours (8A) or 24 hours (8B), n = at least 6 subjects per condition, Mann-Whitney testing. 8c) Percent of macrophages deemed viable per naphthol staining after a 24 hour infection, n = 2.

*B. cenocepacia* promotes increased cell death in human CF macrophages [19]. Autophagy has an interconnected role with apoptosis and necrosis in the regulation of cell death [53]. We examined MDMs for LDH release at 4 and 24 hours after infection to assess cell death as a result of autophagy stimulation in CF macrophages infected with *B. cenocepacia*. After 4 hours of infection IFN-γ treated CF MDMs trended towards higher cell death compared to untreated CF MDMs (Figure 8A). All CF macrophages had higher cell death than non-CF macrophages (Figure 8A). After 24 of infection CF macrophages remained with slightly higher cell death than non-CF, except for IFN-γ treated CF MDMs. There was also no difference among autophagy stimulated CF macrophages compared to un-stimulated CF macrophages (Figures 8B). Macrophage viability was additionally assessed at 24 hours of infection via a naphthol blue/black assay. At 24 hours there was no difference in macrophage viability between CF and non CF macrophage (Figure 8C). Macrophage viability was not decreased with IFN-γ treatment (Figure 8c). Taken together, this data suggests that autophagic clearance of *B. cenocepacia* in CF macrophages decreases cell death in a human model after IFN-γ treatment.

## Discussion

The discovery of new therapeutic classes or novel utilization of existing therapeutics is imperative for CF patients infected with *B. cenocepacia* due to its devastating impact on morbidity and mortality. Here, we demonstrate the effectiveness of autophagy stimulation as a therapeutic class, specifically IFN-γ in clearing *B. cenocepacia* from human CF macrophages. This is a crucial finding considering the mounting evidence for CFTR dysfunction in monocyte-pathogen interactions in CF[54], as well as the ability of *B. cenocepacia* to replicate within macrophages [24], [55]. By improving the clearance of *B. cenocepacia* from macrophages, we can minimize the chances of overwhelming bacteremia as well as reduce continued pro-inflammatory signaling. Our work is aligned well with a recent study from Al-Khodor and colleagues in CGD macrophages [38], and importantly demonstrates the effectiveness of a post infection therapeutic intervention. Autophagy stimulation also holds the promise of clearing other pathogens in CF besides *B. cenocepacia* [27], [28], [56], as well as facilitating CFTR trafficking [30].

IFN-γ has numerous signaling effects in the body including host defense, autoimmunity, inflammatory responses, and is a major activator of macrophages [57]. Importantly, we found deficient production of IFN-γ with adequate receptor expression in CF PBMCs in response to *B. cenocepacia* compared to non-CF PBMCs, suggesting a natural defect in CF macrophage priming/pathogen response. This was in contrast to our previous work with the J2315 strain [19], however our previous study may have been affected by the different strain as well as negligible baseline IFN-γ from pure macrophage cultures. IFN-γ has been previously studied in CF as an inhalational therapy in patients without *Burkholderia* infections, demonstrating no significant effects on lung function, sputum bacterial density, or inflammatory sputum markers [41]. However, in that study only 2 of the 48 treated patients had detected serum IFN-γ levels, and they did not measure lower airway IFN-γ levels to monitor deposition efficacy. Furthermore, the effective aerosol deposition of IFN-γ is low in several non-CF studies, which questions the efficacy of the aforementioned CF study as alveolar macrophages would not be affected by upper airway deposition. A 2004 study of aerosolized IFN-γ in TB patients demonstrated greater upper airway deposition of IFN-γ than lower airway, with a mean lower airway deposition of only 35.8 µg of a 500 ug dose [58]. Recent studies in patients with idiopathic pulmonary fibrosis demonstrated only 65% lower airway deposition using a Philips Respironics I-neb [59]. Based on the sputum density of CF patients it is reasonable to assume that nebulized concentrations of IFN-γ would be even lower in CF patients’ lower airways, necessitating systemic IFN-γ use in situations such as *B. cenocepacia* septicemia or worsening patient progression until improved aerosol deposition can be achieved. Prior studies in CF murine models of *P. aeruginosa* infection effectively utilized IFN-γ as a systemic therapy [60], and patients with CGD inject IFN-γ subcutaneously to prevent infections with species including *Burkholderia* [34]. Known side effects of IFN-γ administration are fever, headaches, myalgias, fatigue, irritability, and flulike syndromes, but overall it has been safely used in CGD [61]. The effects of IFN-γ on the total CF microbiome still remain to be characterized, but on individual pathogens such as *B. cenocepacia* and *P. aeruginosa*, there is a demonstrable benefit on bacterial clearance.

Autophagy stimulation is also known to reduce inflammasome mediated IL-1β production [62] and attenuates hyperinflammatory responses from CF cells [29] independent of *B. cenocepacia.* We demonstrated that IFN-γ was effective in reducing the exaggerated IL-1β production that is observed in response to *B. cenocepacia* in CF macrophages. This result was not unexpected given the possibility of inflammasome dependent IFN-γ signaling [63], as well as the observed reduction in bacteria with IFN-γ treatment in the CF macrophages. Therefore, our results suggest the reductions in IL-1β are likely due to both the reduced burden observed and dampened inflammasome activation. IL-10 was unchanged with treatment, but was not significantly different between CF and non CF prior to treatment, and therefore not likely to be reduced with IFN-γ therapy. Importantly, IL-10 was not significantly overproduced in response to therapy.

In addition to reductions in bacteria and inflammatory cytokines, autophagy stimulation was effective in increasing autophagosome formation and trafficking of *B. cenocepacia* to lysosomes. *B. cenocepacia-*containing vacuoles have been shown to have prolonged arrest phases [64], and the ability to correctly utilize otherwise sequestered autophagic machinery may be key in overcoming this delay. Electron microscopy confirmed the presence of single membrane vacuoles containing *B. cenocepacia* in untreated CF macrophages that were effectively converted to double membrane autophagosomes upon IFN-γ stimulation. This process was marked by decreased p62 accumulation and decreased LC3-I accumulation, suggesting effective autophagic flux, allowing for early autophagolysosomal fusion and subsequent bacterial clearance.

This study is limited by the use of a single pathogen model, which was necessary to accurately follow *B. cenocepacia* trafficking as well as cytokine production. Future studies will examine multi-pathogen models to examine more closely effects on pathogen interactions. Further work is also needed *in vivo* to determine the benefits in human subjects. *B. cenocepacia* also possesses several quorum sensing systems that may affect its activity in humans and during a 24 hour infection model [65]–[67]. This will be important to consider in future multi-pathogen studies. Additionally, we tried to overcome inherent human subject differences in cell signaling and baseline medications through the use of multiple subjects. Importantly, we did not see differences in *B. cenocepacia* clearance by CF patients chronically on azithromycin, but samples did not receive further supplementation with azithromycin during any of the experiments.

In summary, CF macrophages have a deficient IFN-γ response to *B. cenocepacia*, ineffective utilization of the autophagy cargo molecule p62, decreased autophagosome formation, and delayed lysosomal uptake. IFN-γ treatment increased autophagy mediated clearance of *B. cenocepacia* in human CF macrophages via increased autophagosome formation and lysosomal trafficking, and was associated with decreased inflammatory cytokine production. The use of IFN-γ or autophagy stimulating drugs is promising for treating life-threatening or chronic infections from *B. cenocepacia* in patients with CF, but further study is needed to determine their efficacy outside of cell culture models.

## Supporting Information

Figure S1 **Chronic azithromycin use has no effect on CF MDMs.** S1A) Serum (n = 7) and infected PBMC (k56, n = 5) IFN-γ levels from CF and non-CF patients separated by patients with (+azithromycin) and without chronic azithromycin prophylaxis. S1B) Summary of scored bacteria per 100 macrophages from individual subjects for the confocal microscopy experiments from 4B and separated by patients with (+azithromycin) and without chronic azithromycin prophylaxis. S1C) IL-1β levels in macrophage supernatants after infection with k56-2 and either a 24 hour treatment with IFN-γ or a control diluent and separated by patients with (+azithromycin) and without chronic azithromycin prophylaxis.
(TIF)

Figure S2 **No difference in 2 hour or supernatant CFUs.** CFU counts for non-CF and CF macrophages infected with MHK1 for 2 (n = 3) and 24 hour supernatants (n = 3) with or without IFN-γ treatment.
(TIF)

Figure S3 **IFN-γ has no effect on bacterial growth in media devoid of MDMs.** Optical density (OD) of bacteria cultured in LB broth alone (k56-2) versus LB + IFN-γ (IFN-γ) was compared over 24 hours during normal growth conditions at 37° with high amplitude shaking. Inset shows negligible difference at 24 hours in OD.
(TIF)

## References

[1] Grasemann H, Ratjen F (2013) Early lung disease in cystic fibrosis. Lancet Respir Med 1: 148–157. 24429095 10.1016/S2213-2600(13)70026-2

[2] Kreda SM, Davis CW, Rose MC (2012) CFTR, mucins, and mucus obstruction in cystic fibrosis. Cold Spring Harb Perspect Med 2: a009589. 22951447 10.1101/cshperspect.a009589 PMC3426818

[3] Govan JR, Brown PH, Maddison J, Doherty CJ, Nelson JW, et al (1993) Evidence for transmission of Pseudomonas cepacia by social contact in cystic fibrosis. Lancet 342: 15–19. 7686239 10.1016/0140-6736(93)91881-l

[4] Sun L, Jiang RZ, Steinbach S, Holmes A, Campanelli C, et al (1995) The emergence of a highly transmissible lineage of cbl+ Pseudomonas (Burkholderia) cepacia causing CF centre epidemics in North America and Britain. Nat Med 1: 661–666. 7585148 10.1038/nm0795-661

[5] Walsh NM, Casano AA, Manangan LP, Sinkowitz-Cochran RL, Jarvis WR (2002) Risk factors for Burkholderia cepacia complex colonization and infection among patients with cystic fibrosis. J Pediatr 141: 512–517. 12378190 10.1067/mpd.2002.127665

[6] Drevinek P, Mahenthiralingam E (2010) Burkholderia cenocepacia in cystic fibrosis: epidemiology and molecular mechanisms of virulence. Clin Microbiol Infect 16: 821–830. 20880411 10.1111/j.1469-0691.2010.03237.x

[7] Jones AM, Dodd ME, Govan JR, Barcus V, Doherty CJ, et al (2004) Burkholderia cenocepacia and Burkholderia multivorans: influence on survival in cystic fibrosis. Thorax 59: 948–951. 15516469 10.1136/thx.2003.017210 PMC1746874

[8] Isles A, Maclusky I, Corey M, Gold R, Prober C, et al (1984) Pseudomonas cepacia infection in cystic fibrosis: an emerging problem. J Pediatr 104: 206–210. 6420530 10.1016/s0022-3476(84)80993-2

[9] Alexander BD, Petzold EW, Reller LB, Palmer SM, Davis RD, et al (2008) Survival after lung transplantation of cystic fibrosis patients infected with Burkholderia cepacia complex. Am J Transplant 8: 1025–1030. 18318775 10.1111/j.1600-6143.2008.02186.x

[10] De Soyza A, Meachery G, Hester KL, Nicholson A, Parry G, et al (2010) Lung transplantation for patients with cystic fibrosis and Burkholderia cepacia complex infection: a single-center experience. J Heart Lung Transplant 29: 1395–1404. 20810293 10.1016/j.healun.2010.06.007

[11] Egan TM, Detterbeck FC, Mill MR, Bleiweis MS, Aris R, et al (2002) Long term results of lung transplantation for cystic fibrosis. Eur J Cardiothorac Surg 22: 602–609. 12297180 10.1016/s1010-7940(02)00376-7

[12] Murray S, Charbeneau J, Marshall BC, LiPuma JJ (2008) Impact of burkholderia infection on lung transplantation in cystic fibrosis. Am J Respir Crit Care Med 178: 363–371. 18535253 10.1164/rccm.200712-1834OC

[13] Olland A, Falcoz PE, Kessler R, Massard G (2011) Should cystic fibrosis patients infected with Burkholderia cepacia complex be listed for lung transplantation? Interact Cardiovasc Thorac Surg 13: 631–634. 21920934 10.1510/icvts.2011.271874

[14] Brennan S, Sly PD, Gangell CL, Sturges N, Winfield K, et al (2009) Alveolar macrophages and CC chemokines are increased in children with cystic fibrosis. Eur Respir J 34: 655–661. 19386685 10.1183/09031936.00178508

[15] Bruscia EM, Zhang PX, Ferreira E, Caputo C, Emerson JW, et al (2009) Macrophages directly contribute to the exaggerated inflammatory response in cystic fibrosis transmembrane conductance regulator−/− mice. Am J Respir Cell Mol Biol 40: 295–304. 18776130 10.1165/rcmb.2008-0170OC PMC2645527

[16] Bruscia EM, Zhang PX, Satoh A, Caputo C, Medzhitov R, et al (2011) Abnormal trafficking and degradation of TLR4 underlie the elevated inflammatory response in cystic fibrosis. J Immunol 186: 6990–6998. 21593379 10.4049/jimmunol.1100396 PMC3111054

[17] Del Porto P, Cifani N, Guarnieri S, Di Domenico EG, Mariggio MA, et al (2011) Dysfunctional CFTR alters the bactericidal activity of human macrophages against Pseudomonas aeruginosa. PLoS One 6: e19970. 21625641 10.1371/journal.pone.0019970 PMC3097223

[18] Hartl D, Gaggar A, Bruscia E, Hector A, Marcos V, et al (2012) Innate immunity in cystic fibrosis lung disease. J Cyst Fibros 11: 363–382. 22917571 10.1016/j.jcf.2012.07.003

[19] Kopp BT, Abdulrahman BA, Khweek AA, Kumar SB, Akhter A, et al (2012) Exaggerated inflammatory responses mediated by Burkholderia cenocepacia in human macrophages derived from Cystic fibrosis patients. Biochem Biophys Res Commun 424: 221–227. 22728038 10.1016/j.bbrc.2012.06.066 PMC3408781

[20] Vandivier RW, Richens TR, Horstmann SA, deCathelineau AM, Ghosh M, et al (2009) Dysfunctional cystic fibrosis transmembrane conductance regulator inhibits phagocytosis of apoptotic cells with proinflammatory consequences. Am J Physiol Lung Cell Mol Physiol 297: L677–686. 19633071 10.1152/ajplung.00030.2009 PMC2770781

[21] Di A, Brown ME, Deriy LV, Li C, Szeto FL, et al (2006) CFTR regulates phagosome acidification in macrophages and alters bactericidal activity. Nat Cell Biol 8: 933–944. 16921366 10.1038/ncb1456

[22] Abdulrahman BA, Khweek AA, Akhter A, Caution K, Kotrange S, et al (2011) Autophagy stimulation by rapamycin suppresses lung inflammation and infection by Burkholderia cenocepacia in a model of cystic fibrosis. Autophagy 7: 1359–1370. 21997369 10.4161/auto.7.11.17660 PMC3359483

[23] Kotrange S, Kopp B, Akhter A, Abdelaziz D, Abu Khweek A, et al (2011) Burkholderia cenocepacia O polysaccharide chain contributes to caspase-1-dependent IL-1beta production in macrophages. J Leukoc Biol 89: 481–488. 21178113 10.1189/jlb.0910513 PMC3040464

[24] Vergunst AC, Meijer AH, Renshaw SA, O’Callaghan D (2010) Burkholderia cenocepacia creates an intramacrophage replication niche in zebrafish embryos, followed by bacterial dissemination and establishment of systemic infection. Infect Immun 78: 1495–1508. 20086083 10.1128/IAI.00743-09 PMC2849400

[25] Luciani A, Villella VR, Esposito S, Brunetti-Pierri N, Medina D, et al (2010) Defective CFTR induces aggresome formation and lung inflammation in cystic fibrosis through ROS-mediated autophagy inhibition. Nat Cell Biol 12: 863–875. 20711182 10.1038/ncb2090

[26] Abdulrahman BA, Khweek AA, Akhter A, Caution K, Tazi M, et al (2013) Depletion of the ubiquitin-binding adaptor molecule SQSTM1/p62 from macrophages harboring cftr DeltaF508 mutation improves the delivery of Burkholderia cenocepacia to the autophagic machinery. J Biol Chem 288: 2049–2058. 23148214 10.1074/jbc.M112.411728 PMC3548511

[27] Yuan K, Huang C, Fox J, Laturnus D, Carlson E, et al (2012) Autophagy plays an essential role in the clearance of Pseudomonas aeruginosa by alveolar macrophages. J Cell Sci 125: 507–515. 22302984 10.1242/jcs.094573 PMC3283879

[28] Schnaith A, Kashkar H, Leggio SA, Addicks K, Kronke M, et al (2007) Staphylococcus aureus subvert autophagy for induction of caspase-independent host cell death. J Biol Chem 282: 2695–2706. 17135247 10.1074/jbc.M609784200

[29] Mayer ML, Blohmke CJ, Falsafi R, Fjell CD, Madera L, et al (2013) Rescue of Dysfunctional Autophagy Attenuates Hyperinflammatory Responses from Cystic Fibrosis Cells. J Immunol 190: 1227–1238. 23264659 10.4049/jimmunol.1201404

[30] Luciani A, Villella VR, Esposito S, Gavina M, Russo I, et al (2012) Targeting autophagy as a novel strategy for facilitating the therapeutic action of potentiators on DeltaF508 cystic fibrosis transmembrane conductance regulator. Autophagy 8: 1657–1672. 22874563 10.4161/auto.21483 PMC3494594

[31] Hardinger KL, Cornelius LA, Trulock EP 3rd, Brennan DC (2002) Sirolimus-induced leukocytoclastic vasculitis. Transplantation 74: 739–743. 12352895 10.1097/00007890-200209150-00025

[32] Chhajed PN, Dickenmann M, Bubendorf L, Mayr M, Steiger J, et al (2006) Patterns of pulmonary complications associated with sirolimus. Respiration 73: 367–374. 16127266 10.1159/000087945

[33] Fernandez-Boyanapalli R, McPhillips KA, Frasch SC, Janssen WJ, Dinauer MC, et al (2010) Impaired phagocytosis of apoptotic cells by macrophages in chronic granulomatous disease is reversed by IFN-gamma in a nitric oxide-dependent manner. J Immunol 185: 4030–4041. 20805415 10.4049/jimmunol.1001778 PMC4346245

[34] A controlled trial of interferon gamma to prevent infection in chronic granulomatous disease. The International Chronic Granulomatous Disease Cooperative Study Group. N Engl J Med 324: 509–516. 1846940 10.1056/NEJM199102213240801

[35] Errante PR, Frazao JB, Condino-Neto A (2008) The use of interferon-gamma therapy in chronic granulomatous disease. Recent Pat Antiinfect Drug Discov 3: 225–230. 18991804 10.2174/157489108786242378

[36] McPhail LC, DeChatelet LR, Shirley PS, Wilfert C, Johnston RB Jr, et al (1977) Deficiency of NADPH oxidase activity in chronic granulomatous disease. J Pediatr 90: 213–217. 12254 10.1016/s0022-3476(77)80632-x

[37] Sanmun D, Witasp E, Jitkaew S, Tyurina YY, Kagan VE, et al (2009) Involvement of a functional NADPH oxidase in neutrophils and macrophages during programmed cell clearance: implications for chronic granulomatous disease. Am J Physiol Cell Physiol 297: C621–631. 19570889 10.1152/ajpcell.00651.2008

[38] Al-Khodor S, Marshall-Batty K, Nair V, Ding L, Greenberg DE, et al (2014) Burkholderia cenocepacia J2315 escapes to the cytosol and actively subverts autophagy in human macrophages. Cell Microbiol 16: 378–395. 24119232 10.1111/cmi.12223 PMC3927784

[39] Gutierrez MG, Master SS, Singh SB, Taylor GA, Colombo MI, et al (2004) Autophagy is a defense mechanism inhibiting BCG and Mycobacterium tuberculosis survival in infected macrophages. Cell 119: 753–766. 15607973 10.1016/j.cell.2004.11.038

[40] Holland SM (2010) Chronic granulomatous disease. Clin Rev Allergy Immunol 38: 3–10. 19504359 10.1007/s12016-009-8136-z

[41] Moss RB, Mayer-Hamblett N, Wagener J, Daines C, Hale K, et al (2005) Randomized, double-blind, placebo-controlled, dose-escalating study of aerosolized interferon gamma-1b in patients with mild to moderate cystic fibrosis lung disease. Pediatr Pulmonol 39: 209–218. 15573395 10.1002/ppul.20152

[42] Hamad MA, Skeldon AM, Valvano MA (2010) Construction of aminoglycoside-sensitive Burkholderia cenocepacia strains for use in studies of intracellular bacteria with the gentamicin protection assay. Appl Environ Microbiol 76: 3170–3176. 20348312 10.1128/AEM.03024-09 PMC2869153

[43] Akhter A, Gavrilin MA, Frantz L, Washington S, Ditty C, et al (2009) Caspase-7 activation by the Nlrc4/Ipaf inflammasome restricts Legionella pneumophila infection. PLoS Pathog 5: e1000361. 19343209 10.1371/journal.ppat.1000361 PMC2657210

[44] Renna M, Schaffner C, Brown K, Shang S, Tamayo MH, et al (2011) Azithromycin blocks autophagy and may predispose cystic fibrosis patients to mycobacterial infection. J Clin Invest 121: 3554–3563. 21804191 10.1172/JCI46095 PMC3163956

[45] Binder AM, Adjemian J, Olivier KN, Prevots DR (2013) Epidemiology of nontuberculous mycobacterial infections and associated chronic macrolide use among persons with cystic fibrosis. Am J Respir Crit Care Med 188: 807–812. 23927602 10.1164/rccm.201307-1200OC PMC3826274

[46] Al-Zeer MA, Al-Younes HM, Lauster D, Abu Lubad M, Meyer TF (2013) Autophagy restricts Chlamydia trachomatis growth in human macrophages via IFNG-inducible guanylate binding proteins. Autophagy 9: 50–62. 23086406 10.4161/auto.22482 PMC3542218

[47] Matsuzawa T, Kim BH, Shenoy AR, Kamitani S, Miyake M, et al (2012) IFN-gamma elicits macrophage autophagy via the p38 MAPK signaling pathway. J Immunol 189: 813–818. 22675202 10.4049/jimmunol.1102041 PMC3392356

[48] Moser C, Kjaergaard S, Pressler T, Kharazmi A, Koch C, et al (2000) The immune response to chronic Pseudomonas aeruginosa lung infection in cystic fibrosis patients is predominantly of the Th2 type. APMIS 108: 329–335. 10937769 10.1034/j.1600-0463.2000.d01-64.x

[49] Komatsu M, Kageyama S, Ichimura Y (2012) p62/SQSTM1/A170: physiology and pathology. Pharmacol Res 66: 457–462. 22841931 10.1016/j.phrs.2012.07.004

[50] McKeon S, McClean S, Callaghan M (2010) Macrophage responses to CF pathogens: JNK MAP kinase signaling by Burkholderia cepacia complex lipopolysaccharide. FEMS Immunol Med Microbiol 60: 36–43. 20602636 10.1111/j.1574-695X.2010.00712.x

[51] Gillette DD, Shah PA, Cremer T, Gavrilin MA, Besecker BY, et al (2013) Analysis of human bronchial epithelial cell proinflammatory response to Burkholderia cenocepacia infection: inability to secrete il-1beta. J Biol Chem 288: 3691–3695. 23269671 10.1074/jbc.C112.430298 PMC3567624

[52] Gavrilin MA, Abdelaziz DH, Mostafa M, Abdulrahman BA, Grandhi J, et al (2012) Activation of the Pyrin Inflammasome by Intracellular Burkholderia cenocepacia. J Immunol 188: 3469–3477. 22368275 10.4049/jimmunol.1102272 PMC3482472

[53] Jain MV, Paczulla AM, Klonisch T, Dimgba FN, Rao SB, et al (2013) Interconnections between apoptotic, autophagic and necrotic pathways: implications for cancer therapy development. J Cell Mol Med 17: 12–29. 23301705 10.1111/jcmm.12001 PMC3823134

[54] Van de Weert-van Leeuwen PB, Van Meegen MA, Speirs JJ, Pals DJ, Rooijakkers SH, et al (2013) Optimal complement-mediated phagocytosis of Pseudomonas aeruginosa by monocytes is cystic fibrosis transmembrane conductance regulator-dependent. Am J Respir Cell Mol Biol 49: 463–470. 23617438 10.1165/rcmb.2012-0502OC

[55] Sajjan SU, Carmody LA, Gonzalez CF, LiPuma JJ (2008) A type IV secretion system contributes to intracellular survival and replication of Burkholderia cenocepacia. Infect Immun 76: 5447–5455. 18824538 10.1128/IAI.00451-08 PMC2583573

[56] Junkins RD, Shen A, Rosen K, McCormick C, Lin TJ (2013) Autophagy enhances bacterial clearance during P. aeruginosa lung infection. PLoS One 8: e72263. 24015228 10.1371/journal.pone.0072263 PMC3756076

[57] Farrar MA, Schreiber RD (1993) The molecular cell biology of interferon-gamma and its receptor. Annu Rev Immunol 11: 571–611. 8476573 10.1146/annurev.iy.11.040193.003035

[58] Condos R, Hull FP, Schluger NW, Rom WN, Smaldone GC (2004) Regional deposition of aerosolized interferon-gamma in pulmonary tuberculosis. Chest 125: 2146–2155. 15189935 10.1378/chest.125.6.2146

[59] Diaz KT, Skaria S, Harris K, Solomita M, Lau S, et al (2012) Delivery and safety of inhaled interferon-gamma in idiopathic pulmonary fibrosis. J Aerosol Med Pulm Drug Deliv 25: 79–87. 22360317 10.1089/jamp.2011.0919

[60] Johansen HK, Hougen HP, Rygaard J, Hoiby N (1996) Interferon-gamma (IFN-gamma) treatment decreases the inflammatory response in chronic Pseudomonas aeruginosa pneumonia in rats. Clin Exp Immunol 103: 212–218. 8565302 10.1046/j.1365-2249.1996.d01-618.x PMC2200342

[61] Marciano BE, Wesley R, De Carlo ES, Anderson VL, Barnhart LA, et al (2004) Long-term interferon-gamma therapy for patients with chronic granulomatous disease. Clin Infect Dis 39: 692–699. 15356785 10.1086/422993

[62] Shi CS, Shenderov K, Huang NN, Kabat J, Abu-Asab M, et al (2012) Activation of autophagy by inflammatory signals limits IL-1beta production by targeting ubiquitinated inflammasomes for destruction. Nat Immunol 13: 255–263. 22286270 10.1038/ni.2215 PMC4116819

[63] Mitchell AJ, Yau B, McQuillan JA, Ball HJ, Too LK, et al (2012) Inflammasome-dependent IFN-gamma drives pathogenesis in Streptococcus pneumoniae meningitis. J Immunol 189: 4970–4980. 23071286 10.4049/jimmunol.1201687

[64] Lamothe J, Valvano MA (2008) Burkholderia cenocepacia-induced delay of acidification and phagolysosomal fusion in cystic fibrosis transmembrane conductance regulator (CFTR)-defective macrophages. Microbiology 154: 3825–3834. 19047750 10.1099/mic.0.2008/023200-0

[65] Subramoni S, Sokol PA (2012) Quorum sensing systems influence Burkholderia cenocepacia virulence. Future Microbiol 7: 1373–1387. 23231487 10.2217/fmb.12.118

[66] Aubert DF, O’Grady EP, Hamad MA, Sokol PA, Valvano MA (2013) The Burkholderia cenocepacia sensor kinase hybrid AtsR is a global regulator modulating quorum-sensing signalling. Environ Microbiol 15: 372–385. 22830644 10.1111/j.1462-2920.2012.02828.x

[67] O’Grady EP, Viteri DF, Sokol PA (2012) A unique regulator contributes to quorum sensing and virulence in Burkholderia cenocepacia. PLoS One 7: e37611. 22624054 10.1371/journal.pone.0037611 PMC3356288

