# Correction: IFN-*γ* Stimulates Autophagy-Mediated Clearance of *Burkholderia cenocepacia* in Human Cystic Fibrosis Macrophages

**DOI:** 10.1371/journal.pone.0213092

**Published:** 2019-02-26

**Authors:** Kaivon Assani, Mia F. Tazi, Amal O. Amer, Benjamin T. Kopp

After publication of this article [1], concerns were raised about image irregularities in Fig 2C, specifically that the Non CF Beclin-1 panel and the CF Beclin-1 panel appear to be taken from the same image. The authors acknowledge that an error was made in generating Fig 2C of this article. In Fig 2C, the Western blot image of Beclin-1 non-CF was inadvertently shown for both the CF and non-CF lanes. We have corrected this error and the updated Fig 2C shows the correct Beclin-1 blots for both CF and non-CF from the original experiments at the original exposure. The corrected Beclin-1 blot does not change the original conclusions for this figure. Please see the correct Fig 2 here.

**Fig 2:**
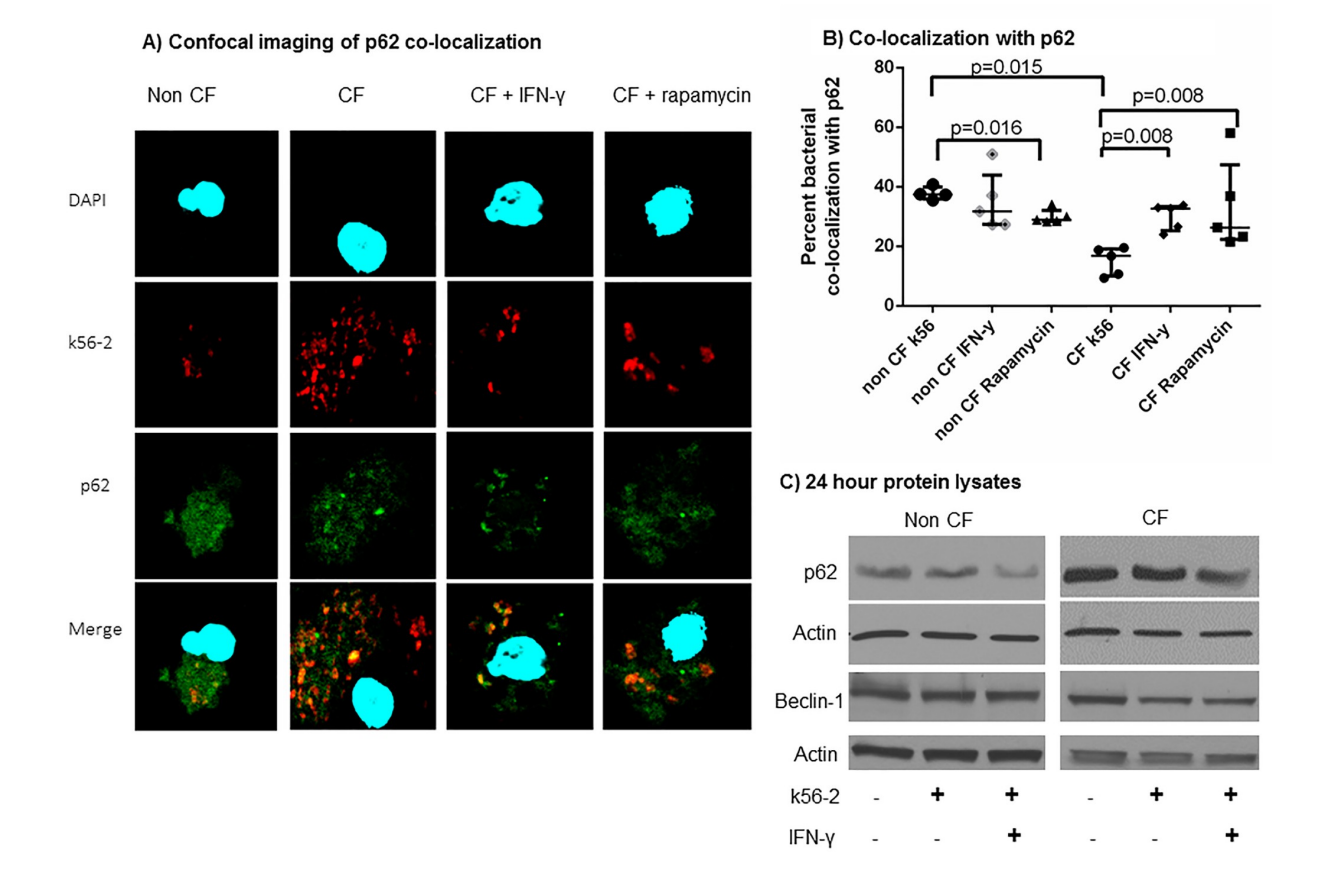
IFN-γ increases *B*. *cenocepacia* co-localization with p62 and decreases p62 accumulation in CF. 2A) Confocal microscopy for non-CF and CF macrophages infected with m-RFP expressing k56-2. IFN-y or rapamycin treatment was administered after 1 hour of infection for a 24 hour treatment period. p62 is stained green, and macrophage nuclei are stained blue with DAPI. Co-localization of bacteria with p62 is noted in yellow in the bottom panel. 2B) The percentage of bacterial co-localization with p62 was scored for over 100 macrophages per condition, n  =  5 subjects per condition, Mann-Whitney testing. 2C) Immunoblot for non-CF and CF macrophages demonstrating p62 accumulation in CF with reduction during IFN-y therapy, representative of 5 subjects. Immunoblot of beclin-1 levels for non-CF and CF macrophages from cell lysates of control (NT) and MDMs infected with k56-2+/− treatment with IFN-γ, n  =  4.

Additionally, we have provided a new Supporting Information file, S1 File, showing one of the other representative independent Western blots to support the CF data in Fig 2C. With this Correction, the authors also provide the original raw data for Fig 2C (in S2–S4 Files), as well as the original data for Fig 2A and 2B (in S5 and S6 Files). All proteins were normalized to their loading controls. Please view Supporting Information files S1–S6 Files below.

A member of *PLOS ONE*’s Editorial Board confirmed that the new results support the results and conclusions of the published article.

The authors apologize for the error in the published article.

## Supporting information

S1 File Supporting experiment for Fig 2C. Representative immunoblot of replicate data for Fig 2C. Immunoblot for non-CF and CF macrophages demonstrating p62 accumulation in CF with reduction during IFN-y therapy. Immunoblot of beclin-1 levels for non-CF and CF macrophages from cell lysates of control (NT) and MDMs infected with k56-2+/− treatment with IFN-γ, with no change during treatment.
(TIF)

S2 File Fig 2 original beclin-1 immunoblots. Original beclin-1 immunoblots for independent experiments used in Fig 2C. Immunoblot of beclin-1 levels for non-CF and CF macrophages from cell lysates of control (NT) and MDMs infected with k56-2+/− treatment with IFN-γ. Other experimental conditions not presented in the manuscript are also present.
(TIF)

S3 File Fig 2 original p62 immunoblots. Original p62 immunoblots for independent experiments used in Fig 2C. Immunoblot for non-CF and CF macrophages demonstrating p62 accumulation in CF with reduction during IFN-y therapy. Other experimental conditions not presented in the manuscript are also present.
(TIF)

S4 File Fig 2 original loading controls. Original loading control immunoblots for independent experiments used in Fig 2C. Other experimental conditions not presented in the manuscript are also present.
(TIF)

S5 File Fig 2A images. Original images of fluorescent channels used for Fig 2A. Each condition has an overlay, DAPI, infection denoted with RFP-expressing bacteria, and p62 detected by GFP-antibody.
(ZIP)

S6 File Fig 2B raw data. Raw data used to create Fig 2B. Percent co-localization of bacteria with p62 per 100 macrophages are shown for each condition. Raw data was used in GraphPad Prism to create Fig 2B.
(XLSX)
