# Supplementary figures and images for: IFN-γ Stimulates Autophagy-Mediated Clearance of Burkholderia cenocepacia in Human Cystic Fibrosis Macrophages
Source: PLoS One. 2014 May 5;9(5):e96681. doi: 10.1371/journal.pone.0096681 (PMC4010498; doi:10.1371/journal.pone.0096681)

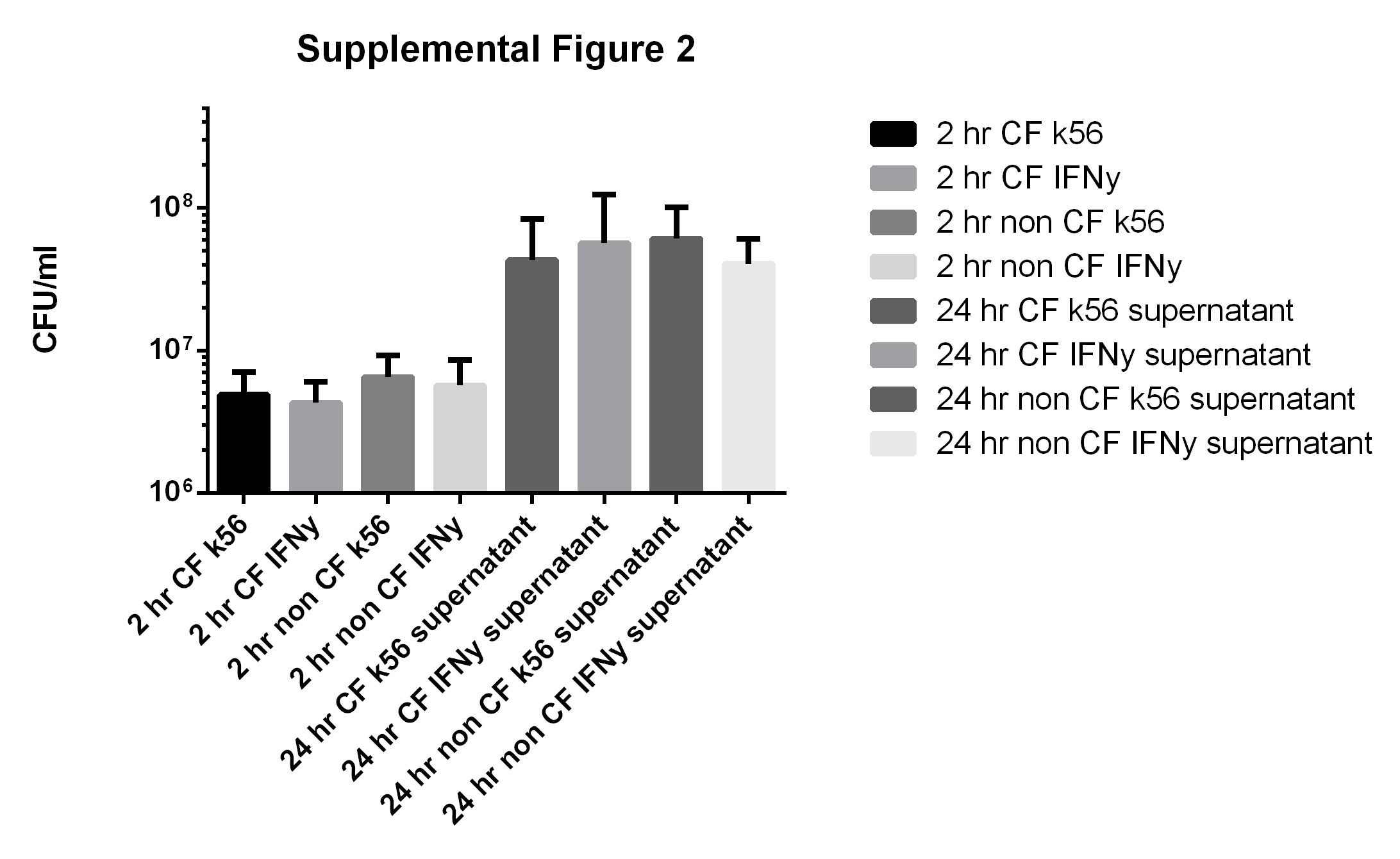

Supplement: Figure S2 — No difference in 2 hour or supernatant CFUs. CFU counts for non-CF and CF macrophages infected with MHK1 for 2 (n = 3) and 24 hour supernatants (n = 3) with or without IFN-γ treatment. (TIF) [file pone.0096681.s002.tif]

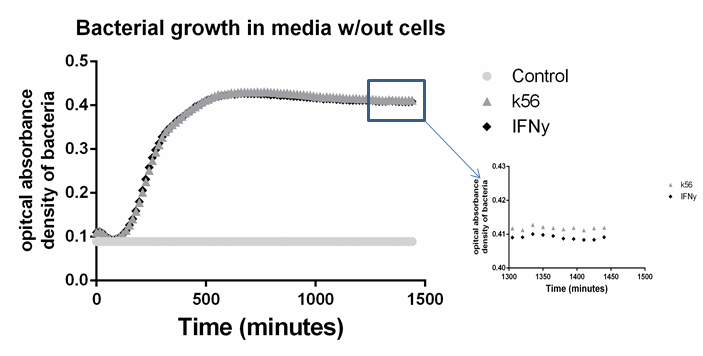

Supplement: Figure S3 — IFN-γ has no effect on bacterial growth in media devoid of MDMs. Optical density (OD) of bacteria cultured in LB broth alone (k56-2) versus LB + IFN-γ (IFN-γ) was compared over 24 hours during normal growth conditions at 37° with high amplitude shaking. Inset shows negligible difference at 24 hours in OD. (TIF) [file pone.0096681.s003.tif]
