# Supplementary figures and images for: Correction: IFN-γ Stimulates Autophagy-Mediated Clearance of Burkholderia cenocepacia in Human Cystic Fibrosis Macrophages
Source: PLoS One. 2019 Feb 26;14(2):e0213092. doi: 10.1371/journal.pone.0213092 (PMC6390998; doi:10.1371/journal.pone.0213092)

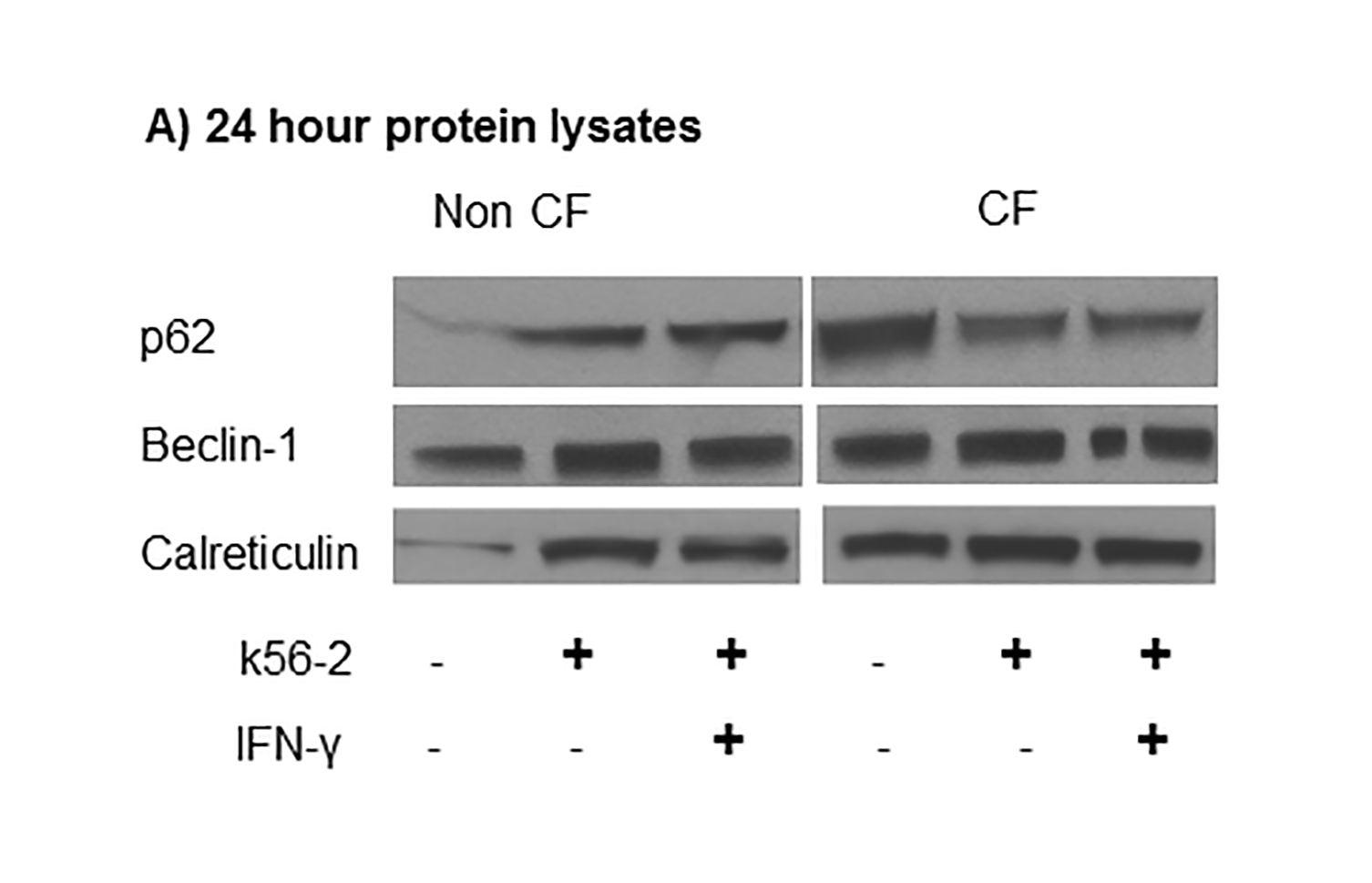

Supplement: S1 File — Representative immunoblot of replicate data for Fig 2C. Immunoblot for non-CF and CF macrophages demonstrating p62 accumulation in CF with reduction during IFN-y therapy. Immunoblot of beclin-1 levels for non-CF and CF macrophages from cell lysates of control (NT) and MDMs infected with k56-2+/− treatment with IFN-γ, with no change during treatment. (TIF) [file pone.0213092.s001.tif]

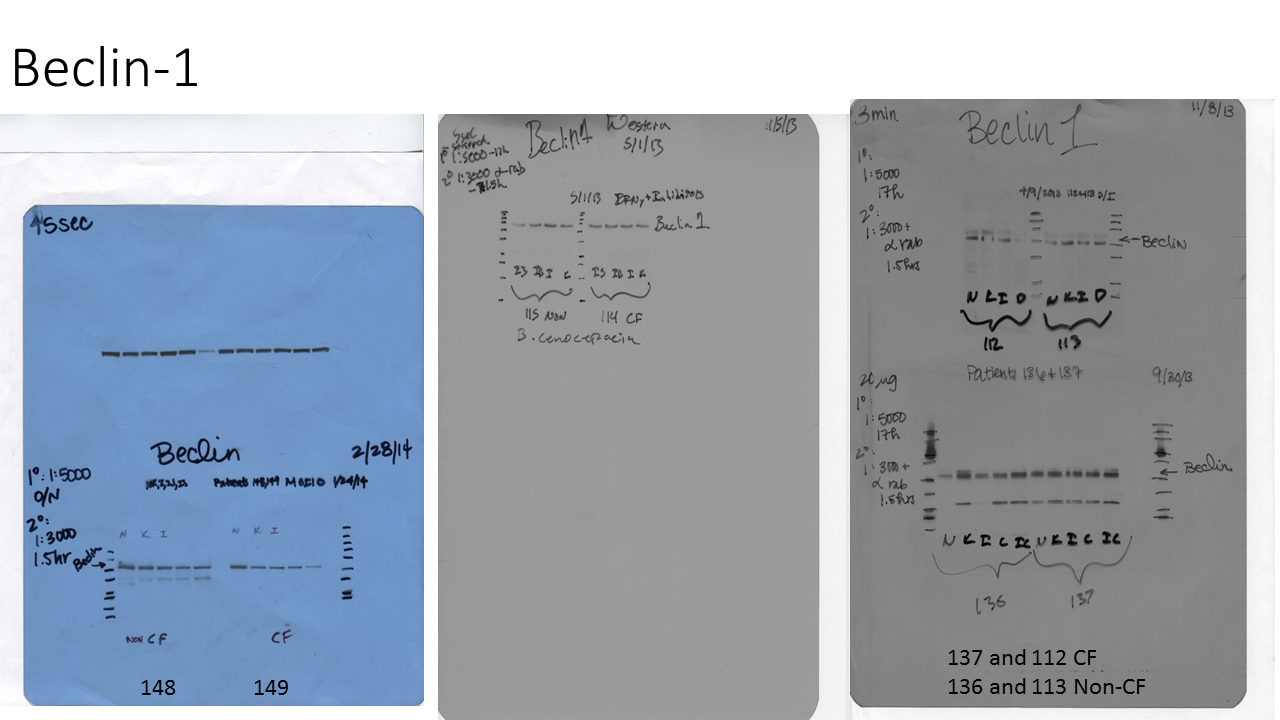

Supplement: S2 File — Original beclin-1 immunoblots for independent experiments used in Fig 2C. Immunoblot of beclin-1 levels for non-CF and CF macrophages from cell lysates of control (NT) and MDMs infected with k56-2+/− treatment with IFN-γ. Other experimental conditions not presented in the manuscript are also present. (TIF) [file pone.0213092.s002.tif]

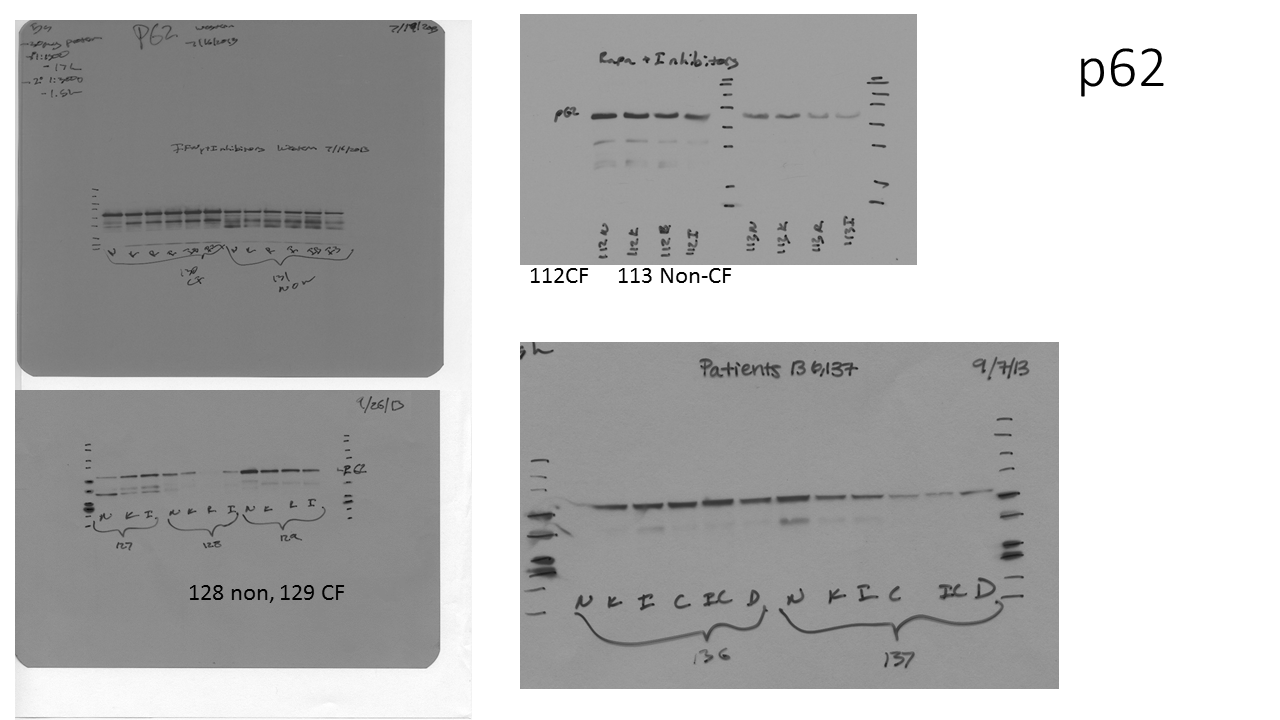

Supplement: S3 File — Original p62 immunoblots for independent experiments used in Fig 2C. Immunoblot for non-CF and CF macrophages demonstrating p62 accumulation in CF with reduction during IFN-y therapy. Other experimental conditions not presented in the manuscript are also present. (TIF) [file pone.0213092.s003.tif]

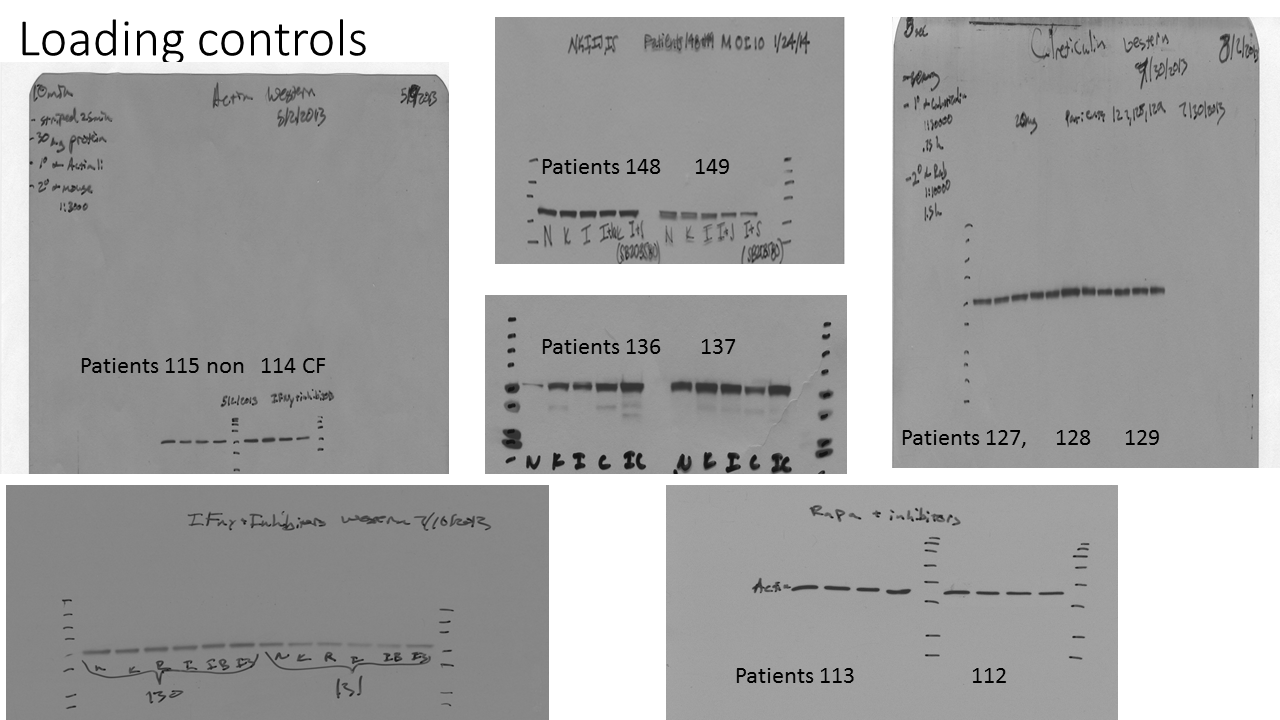

Supplement: S4 File — Original loading control immunoblots for independent experiments used in Fig 2C. Other experimental conditions not presented in the manuscript are also present. (TIF) [file pone.0213092.s004.tif]

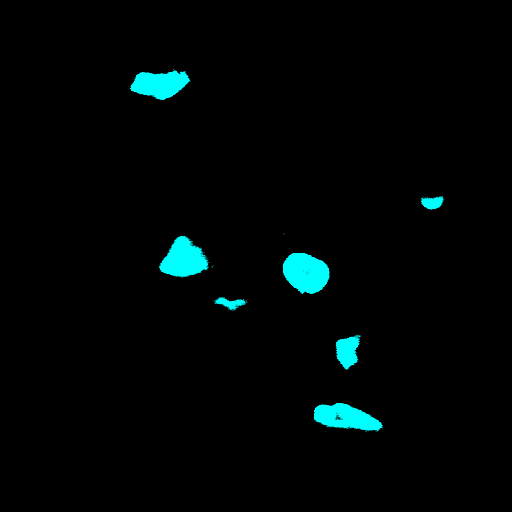

Supplement: S5 File — Original images of fluorescent channels used for Fig 2A. Each condition has an overlay, DAPI, infection denoted with RFP-expressing bacteria, and p62 detected by GFP-antibody. (ZIP) [file pone.0213092.s005.zip › p62/100 CF k56 dapi.bmp]

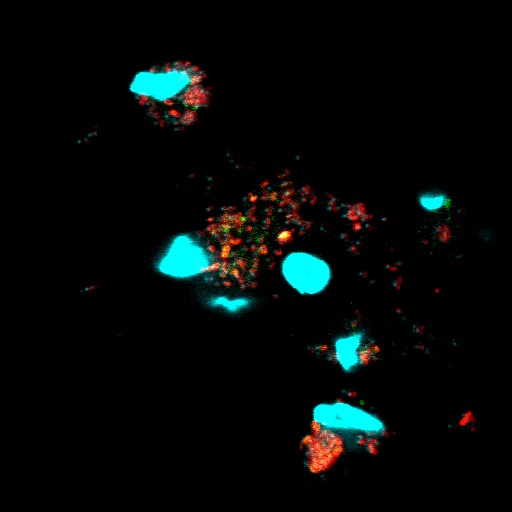

Supplement: S5 File — Original images of fluorescent channels used for Fig 2A. Each condition has an overlay, DAPI, infection denoted with RFP-expressing bacteria, and p62 detected by GFP-antibody. (ZIP) [file pone.0213092.s005.zip › p62/100 CF k56 overlay.bmp]

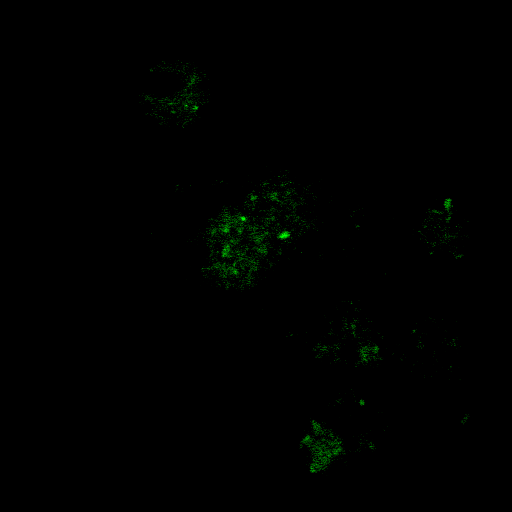

Supplement: S5 File — Original images of fluorescent channels used for Fig 2A. Each condition has an overlay, DAPI, infection denoted with RFP-expressing bacteria, and p62 detected by GFP-antibody. (ZIP) [file pone.0213092.s005.zip › p62/100 CF k56 p62 green.bmp]

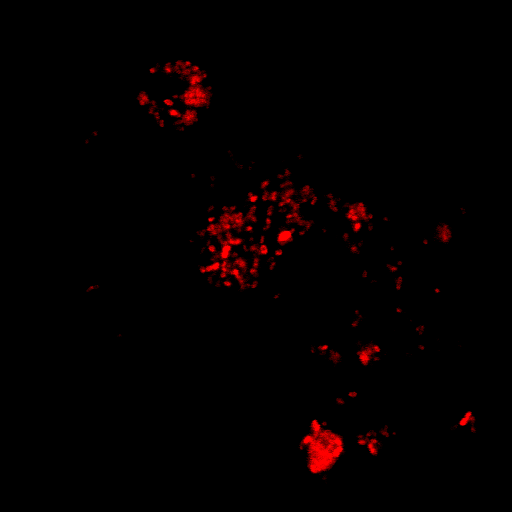

Supplement: S5 File — Original images of fluorescent channels used for Fig 2A. Each condition has an overlay, DAPI, infection denoted with RFP-expressing bacteria, and p62 detected by GFP-antibody. (ZIP) [file pone.0213092.s005.zip › p62/100 CF k56 red.bmp]

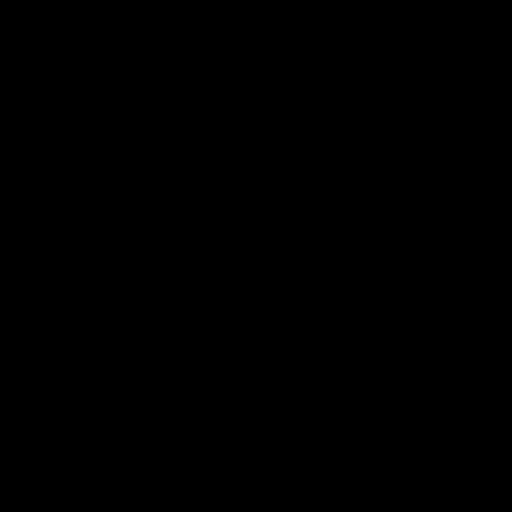

Supplement: S5 File — Original images of fluorescent channels used for Fig 2A. Each condition has an overlay, DAPI, infection denoted with RFP-expressing bacteria, and p62 detected by GFP-antibody. (ZIP) [file pone.0213092.s005.zip › p62/100 CF NT k56 blank.bmp]

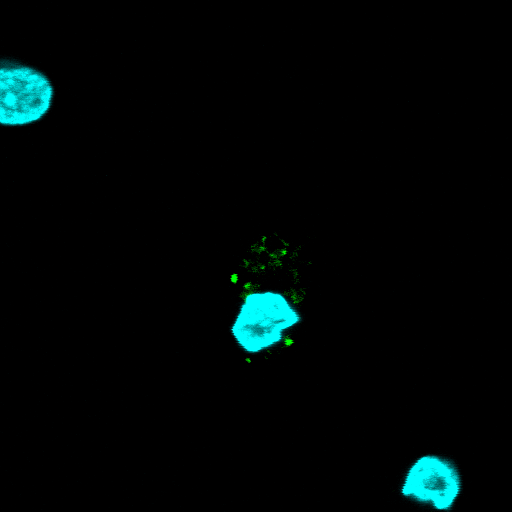

Supplement: S5 File — Original images of fluorescent channels used for Fig 2A. Each condition has an overlay, DAPI, infection denoted with RFP-expressing bacteria, and p62 detected by GFP-antibody. (ZIP) [file pone.0213092.s005.zip › p62/100 CF NT overlay.bmp]

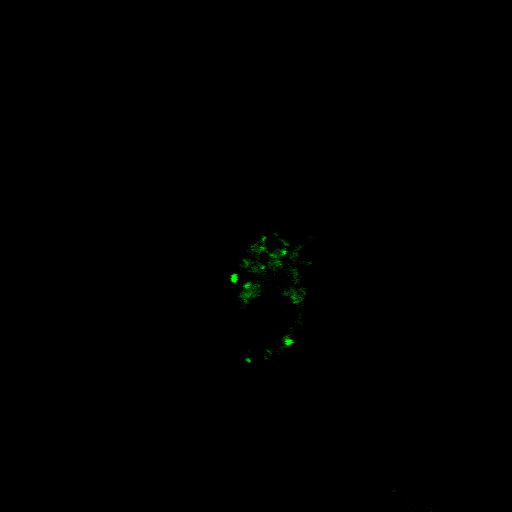

Supplement: S5 File — Original images of fluorescent channels used for Fig 2A. Each condition has an overlay, DAPI, infection denoted with RFP-expressing bacteria, and p62 detected by GFP-antibody. (ZIP) [file pone.0213092.s005.zip › p62/100 CF NT p62.bmp]

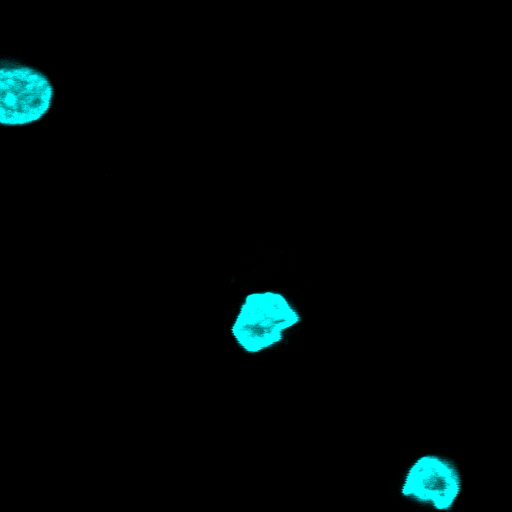

Supplement: S5 File — Original images of fluorescent channels used for Fig 2A. Each condition has an overlay, DAPI, infection denoted with RFP-expressing bacteria, and p62 detected by GFP-antibody. (ZIP) [file pone.0213092.s005.zip › p62/100 CF NTp62 dapi.bmp]

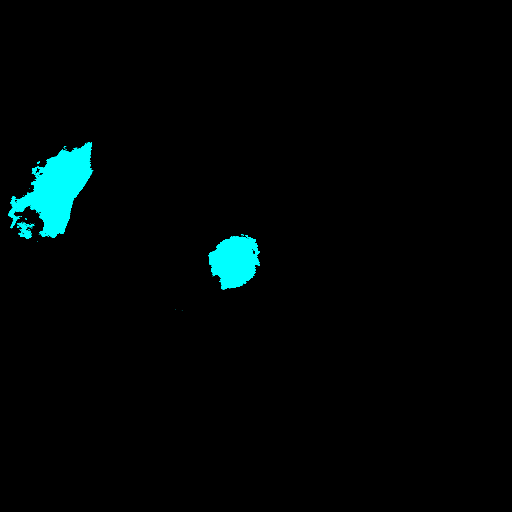

Supplement: S5 File — Original images of fluorescent channels used for Fig 2A. Each condition has an overlay, DAPI, infection denoted with RFP-expressing bacteria, and p62 detected by GFP-antibody. (ZIP) [file pone.0213092.s005.zip › p62/100 cf rapa dapi.bmp]

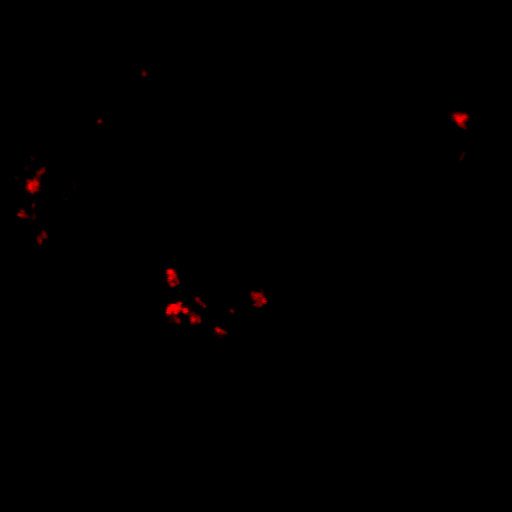

Supplement: S5 File — Original images of fluorescent channels used for Fig 2A. Each condition has an overlay, DAPI, infection denoted with RFP-expressing bacteria, and p62 detected by GFP-antibody. (ZIP) [file pone.0213092.s005.zip › p62/100 cf rapa k56red.bmp]

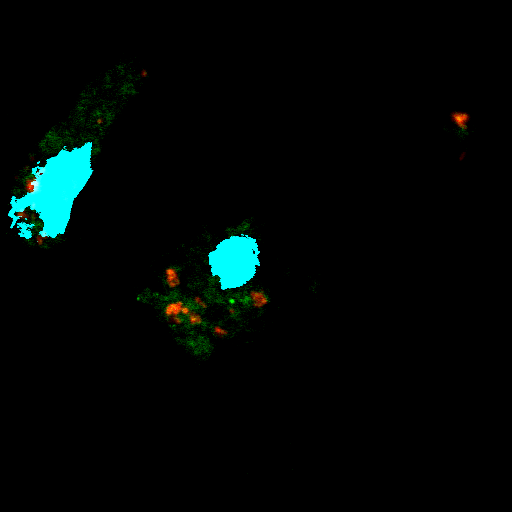

Supplement: S5 File — Original images of fluorescent channels used for Fig 2A. Each condition has an overlay, DAPI, infection denoted with RFP-expressing bacteria, and p62 detected by GFP-antibody. (ZIP) [file pone.0213092.s005.zip › p62/100 cf rapa overlay.bmp]

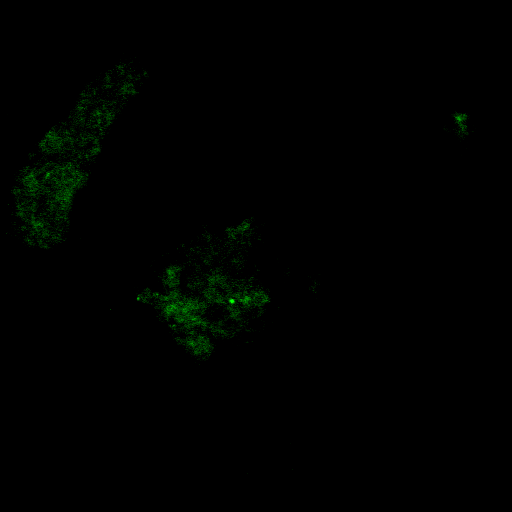

Supplement: S5 File — Original images of fluorescent channels used for Fig 2A. Each condition has an overlay, DAPI, infection denoted with RFP-expressing bacteria, and p62 detected by GFP-antibody. (ZIP) [file pone.0213092.s005.zip › p62/100 cf rapa p62green.bmp]

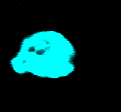

Supplement: S5 File — Original images of fluorescent channels used for Fig 2A. Each condition has an overlay, DAPI, infection denoted with RFP-expressing bacteria, and p62 detected by GFP-antibody. (ZIP) [file pone.0213092.s005.zip › p62/100 IFNy dapi.bmp]

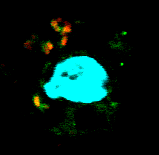

Supplement: S5 File — Original images of fluorescent channels used for Fig 2A. Each condition has an overlay, DAPI, infection denoted with RFP-expressing bacteria, and p62 detected by GFP-antibody. (ZIP) [file pone.0213092.s005.zip › p62/100 IFNy overlay.bmp]

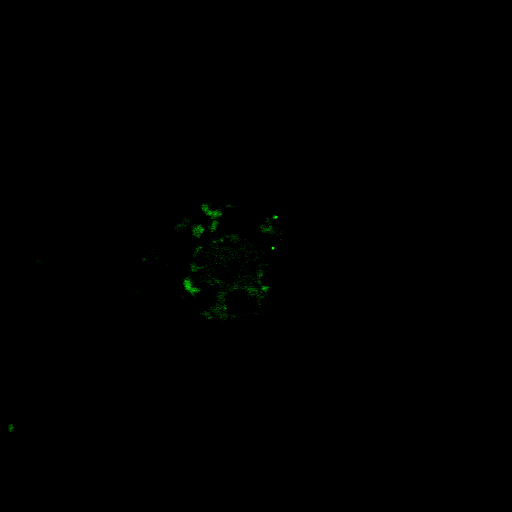

Supplement: S5 File — Original images of fluorescent channels used for Fig 2A. Each condition has an overlay, DAPI, infection denoted with RFP-expressing bacteria, and p62 detected by GFP-antibody. (ZIP) [file pone.0213092.s005.zip › p62/100 IFNy p62 green.bmp]

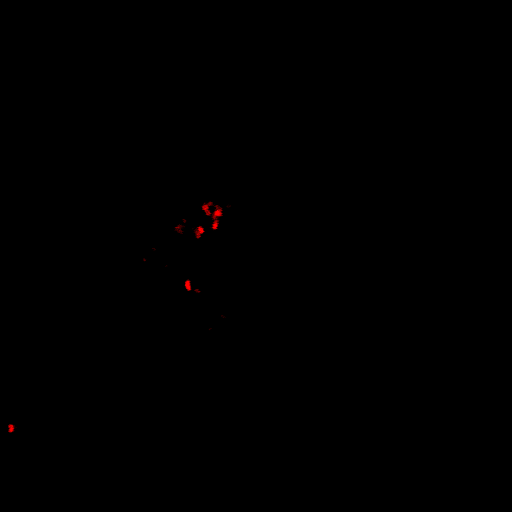

Supplement: S5 File — Original images of fluorescent channels used for Fig 2A. Each condition has an overlay, DAPI, infection denoted with RFP-expressing bacteria, and p62 detected by GFP-antibody. (ZIP) [file pone.0213092.s005.zip › p62/100 IFNy red.bmp]

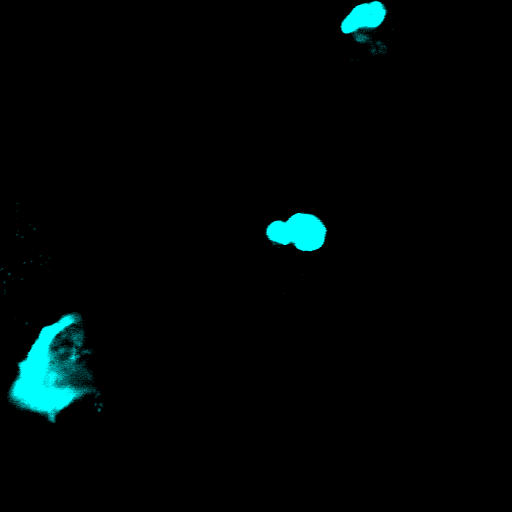

Supplement: S5 File — Original images of fluorescent channels used for Fig 2A. Each condition has an overlay, DAPI, infection denoted with RFP-expressing bacteria, and p62 detected by GFP-antibody. (ZIP) [file pone.0213092.s005.zip › p62/101 non CF k56 dapi.bmp]

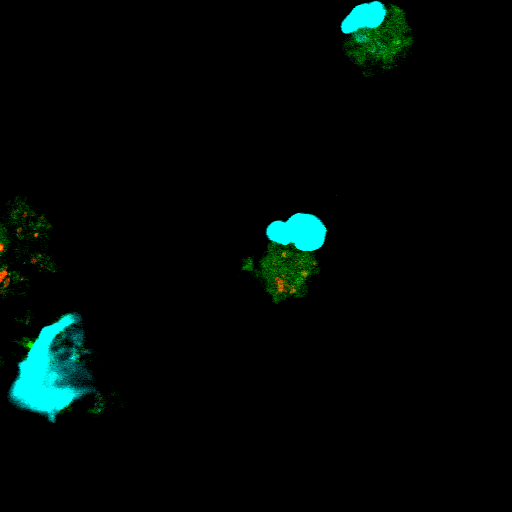

Supplement: S5 File — Original images of fluorescent channels used for Fig 2A. Each condition has an overlay, DAPI, infection denoted with RFP-expressing bacteria, and p62 detected by GFP-antibody. (ZIP) [file pone.0213092.s005.zip › p62/101 non CF k56 overlap.bmp]

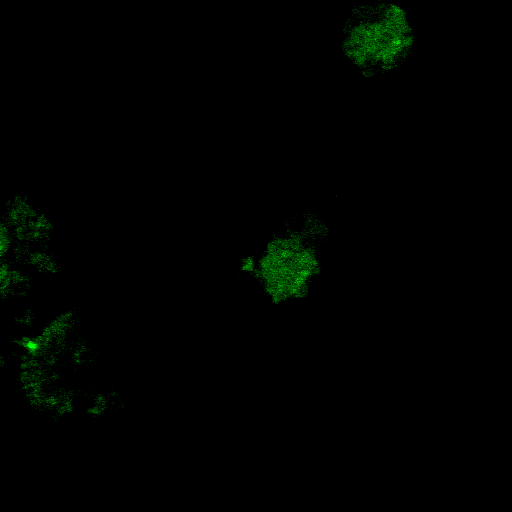

Supplement: S5 File — Original images of fluorescent channels used for Fig 2A. Each condition has an overlay, DAPI, infection denoted with RFP-expressing bacteria, and p62 detected by GFP-antibody. (ZIP) [file pone.0213092.s005.zip › p62/101 non CF k56 p62green.bmp]

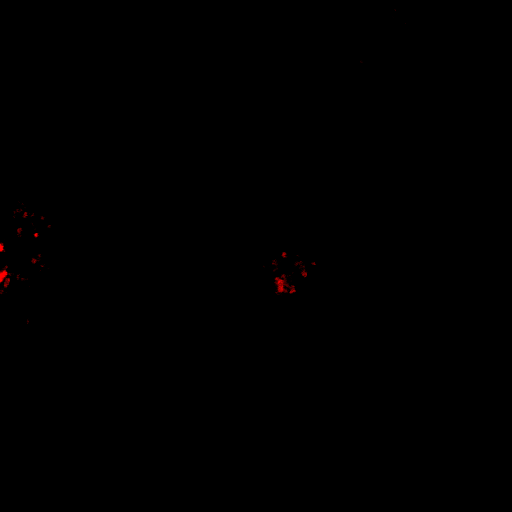

Supplement: S5 File — Original images of fluorescent channels used for Fig 2A. Each condition has an overlay, DAPI, infection denoted with RFP-expressing bacteria, and p62 detected by GFP-antibody. (ZIP) [file pone.0213092.s005.zip › p62/101 non CF k56 red.bmp]

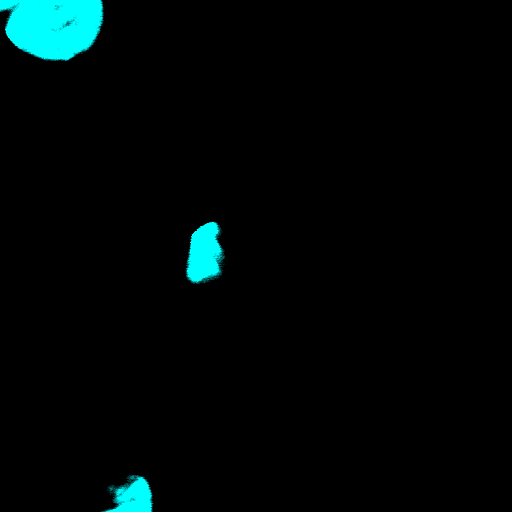

Supplement: S5 File — Original images of fluorescent channels used for Fig 2A. Each condition has an overlay, DAPI, infection denoted with RFP-expressing bacteria, and p62 detected by GFP-antibody. (ZIP) [file pone.0213092.s005.zip › p62/101 non cf nt dapi.bmp]

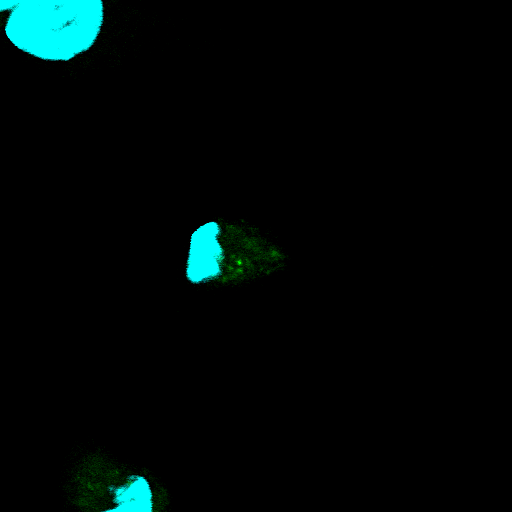

Supplement: S5 File — Original images of fluorescent channels used for Fig 2A. Each condition has an overlay, DAPI, infection denoted with RFP-expressing bacteria, and p62 detected by GFP-antibody. (ZIP) [file pone.0213092.s005.zip › p62/101 non cf nt overlap.bmp]

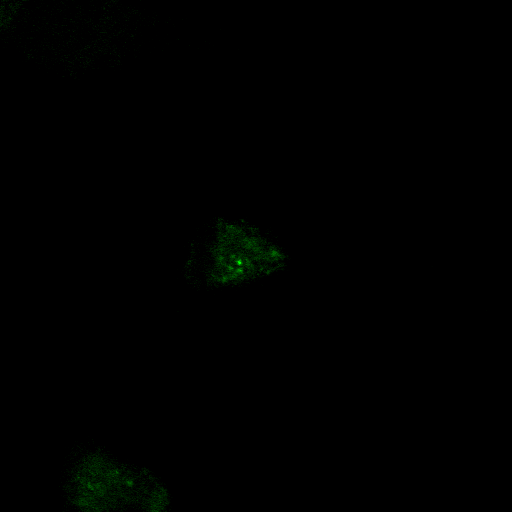

Supplement: S5 File — Original images of fluorescent channels used for Fig 2A. Each condition has an overlay, DAPI, infection denoted with RFP-expressing bacteria, and p62 detected by GFP-antibody. (ZIP) [file pone.0213092.s005.zip › p62/101 non cf nt p62 green.bmp]

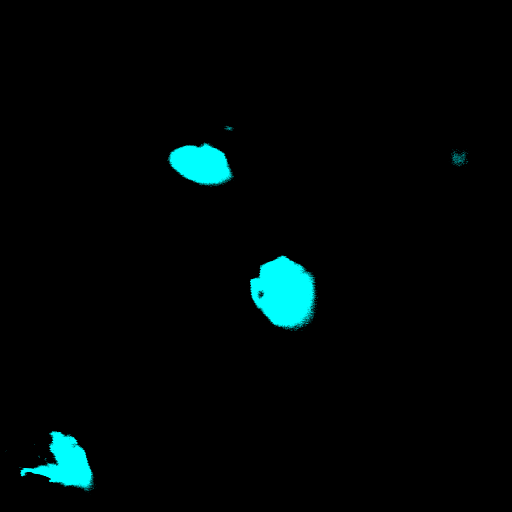

Supplement: S5 File — Original images of fluorescent channels used for Fig 2A. Each condition has an overlay, DAPI, infection denoted with RFP-expressing bacteria, and p62 detected by GFP-antibody. (ZIP) [file pone.0213092.s005.zip › p62/101 non rapa dapi.bmp]

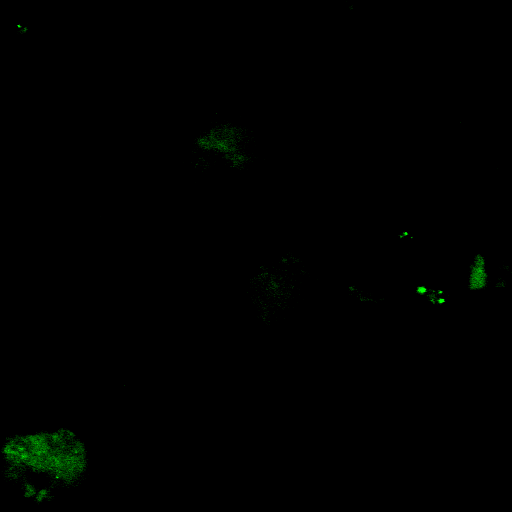

Supplement: S5 File — Original images of fluorescent channels used for Fig 2A. Each condition has an overlay, DAPI, infection denoted with RFP-expressing bacteria, and p62 detected by GFP-antibody. (ZIP) [file pone.0213092.s005.zip › p62/101 non rapa p62 green.bmp]

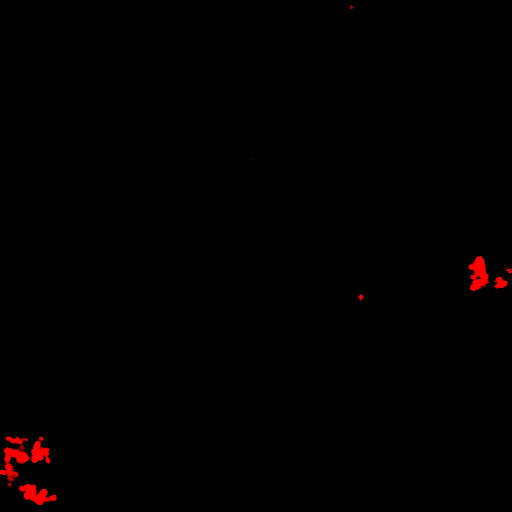

Supplement: S5 File — Original images of fluorescent channels used for Fig 2A. Each condition has an overlay, DAPI, infection denoted with RFP-expressing bacteria, and p62 detected by GFP-antibody. (ZIP) [file pone.0213092.s005.zip › p62/101 non rapa red k56.bmp]

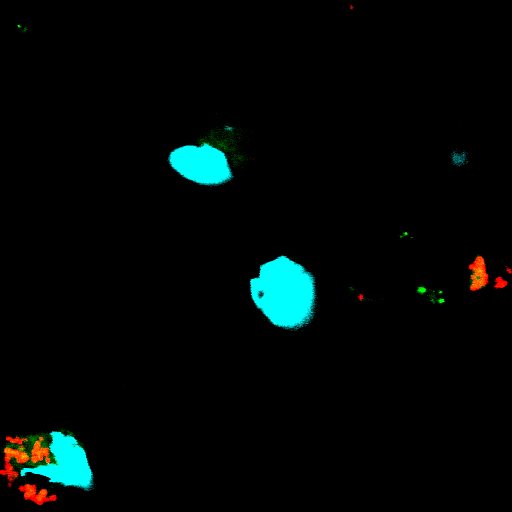

Supplement: S5 File — Original images of fluorescent channels used for Fig 2A. Each condition has an overlay, DAPI, infection denoted with RFP-expressing bacteria, and p62 detected by GFP-antibody. (ZIP) [file pone.0213092.s005.zip › p62/101 non rapaoverlay.bmp]

## Slide 1
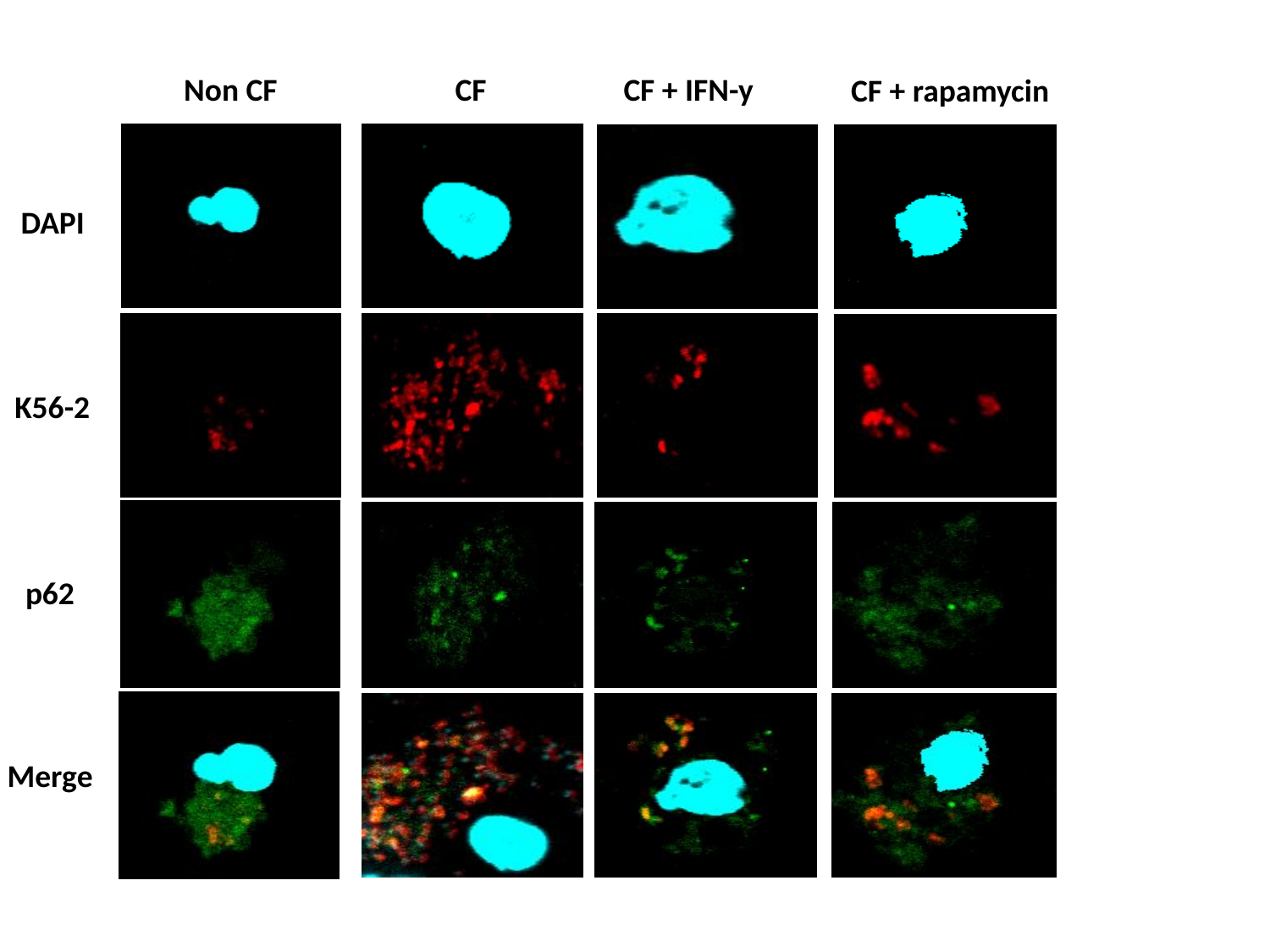

Non CF
CF
CF + rapamycin
CF + IFN-y
DAPI
K56-2
p62
Merge

Supplement: S5 File — Original images of fluorescent channels used for Fig 2A. Each condition has an overlay, DAPI, infection denoted with RFP-expressing bacteria, and p62 detected by GFP-antibody. (ZIP) [file pone.0213092.s005.zip › p62/p62 ifny.pptx]

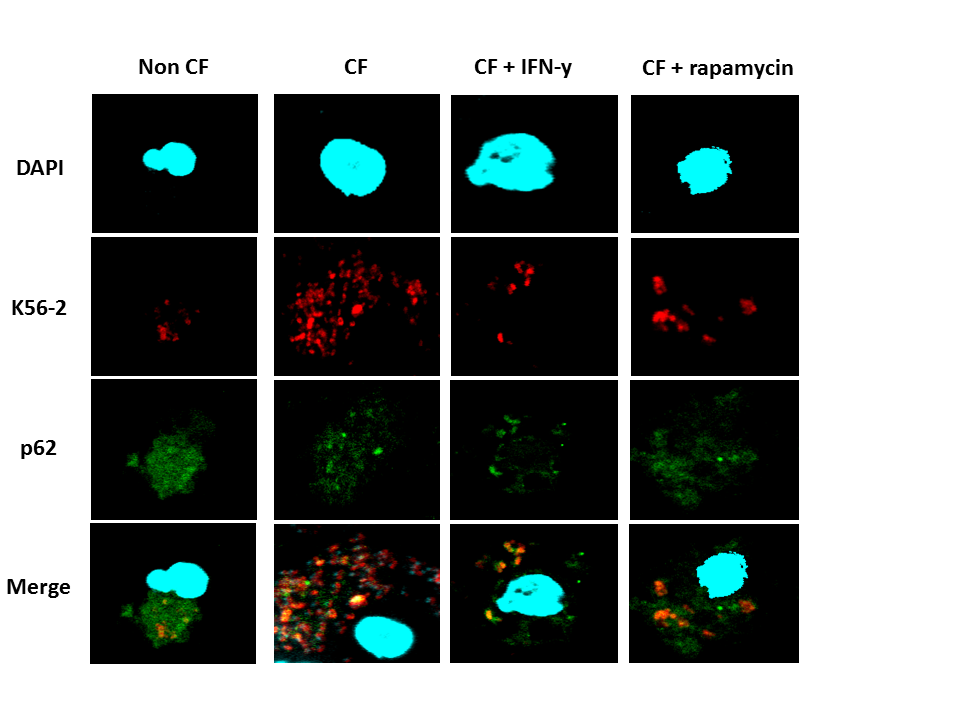

Supplement: S5 File — Original images of fluorescent channels used for Fig 2A. Each condition has an overlay, DAPI, infection denoted with RFP-expressing bacteria, and p62 detected by GFP-antibody. (ZIP) [file pone.0213092.s005.zip › p62/p62 ifny.tif]
